# Quercetin Protects *Saccharomyces cerevisiae* against Oxidative Stress by Inducing Trehalose Biosynthesis and the Cell Wall Integrity Pathway

**DOI:** 10.1371/journal.pone.0045494

**Published:** 2012-09-18

**Authors:** Rita Vilaça, Vanda Mendes, Marta Vaz Mendes, Laura Carreto, Maria Amélia Amorim, Victor de Freitas, Pedro Moradas-Ferreira, Nuno Mateus, Vítor Costa

**Affiliations:** 1 Instituto de Biologia Molecular e Celular, Universidade do Porto, Porto, Portugal; 2 Departamento de Biologia Molecular, Instituto de Ciências Biomédicas Abel Salazar, Universidade do Porto, Porto, Portugal; 3 Centro de Investigação em Química, Faculdade de Ciências da Universidade do Porto, Porto, Portugal; 4 Departamento de Biologia e Centro de Estudos do Ambiente e do Mar, Universidade de Aveiro, Aveiro, Portugal; Instituto de Química - Universidade de São Paulo, Brazil

## Abstract

**Background:**

Quercetin is a naturally occurring flavonol with antioxidant, anticancer and anti-ageing properties. In this study we aimed to identify genes differentially expressed in yeast cells treated with quercetin and its role in oxidative stress protection.

**Methods:**

A microarray analysis was performed to characterize changes in the transcriptome and the expression of selected genes was validated by RT-qPCR. Biological processes significantly affected were identified by using the FUNSPEC software and their relevance in H_2_O_2_ resistance induced by quercetin was assessed.

**Results:**

Genes associated with RNA metabolism and ribosome biogenesis were down regulated in cells treated with quercetin, whereas genes associated with carbohydrate metabolism, endocytosis and vacuolar proteolysis were up regulated. The induction of genes related to the metabolism of energy reserves, leading to the accumulation of the stress protectant disaccharide trehalose, and the activation of the cell wall integrity pathway play a key role in oxidative stress resistance induced by quercetin.

**Conclusions:**

These results suggest that quercetin may act as a modulator of cell signaling pathways related to carbohydrate metabolism and cell integrity to exert its protective effects against oxidative stress.

## Introduction

Oxidative stress is a common hallmark in the genesis of multiple age-associated diseases, such as cardiovascular diseases [1], cancer [2] and neurodegenerative [3] disorders. Oxidative stress is characterized by an imbalance between the production of reactive oxygen species (ROS) or reactive nitrogen species and cellular antioxidant defenses, resulting in the deregulation of redox homeostasis and accumulation of oxidatively damaged proteins, lipids and DNA that may lead to cell death [4]. ROS, such as hydrogen peroxide, superoxide and hydroxyl radicals, are normal by-products of mitochondrial respiration and reactions of cellular metabolism (e.g., catalyzed by cytochrome P450 and flavoprotein oxidases) or are generated from environmental insults. Reactive nitrogen species include nitric oxide (NO) produced by nitric oxide synthases, peroxinitrite generated by nonenzymatic reaction of NO with superoxide radicals, and other species such as nitrogen dioxide and dinitrogen trioxide. To maintain redox homeostasis, cells possess antioxidant defenses that neutralize reactive species in excess and repair oxidative damages [4], [5].

**Figure 1:**
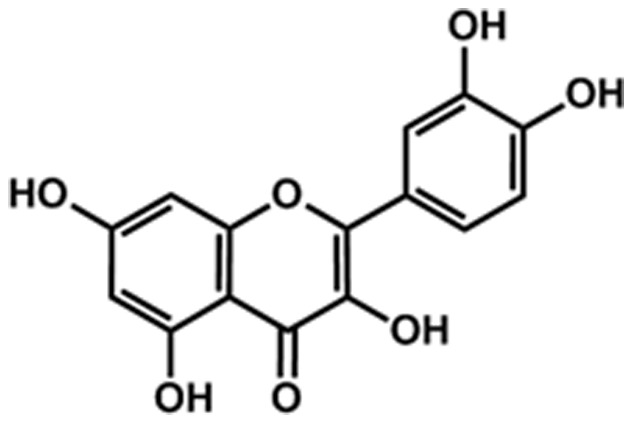
Chemical structure of quercetin.

Epidemiological studies have shown an inverse correlation between the consumption of polyphenol-rich foods and oxidative stress-related chronic diseases [6]. Polyphenols are a group of plant secondary metabolites featuring more than one phenolic ring and without any nitrogen-based functional group in its structure [7]. According to their structure, polyphenols can be divided into different classes, in which flavonoids are the largest class. Quercetin (IUPAC nomenclature: 3,3′,4′,5,7-pentahydroxyflavanone) is a flavonol, a major widespread sub-class of flavonoids, being ubiquitously found in the human diet in onions, shallots, apples, berries, grapes, cappers, brassica vegetables, tea and also in red wine [8]. Quercetin has been extensively studied in many biological models, such as the nematode *Caenorhabditis elegans* [9], mammalian cell models [10], mice [11] and rats [12], and also to some extend in humans [13]. It is known to exhibit numerous biological and pharmacological effects, owning to its antioxidant properties [14], anti-inflammatory [15], and anticarcinogenic [16] effects. The intrinsic antioxidant activities of quercetin have been attributed to direct scavenging of ROS, through the abstraction of unpaired electrons or hydrogen atoms, or metal ions chelation that prevents the generation of hydroxyl radical through Fenton-type reactions. These properties are largely a function of the chemical structure of quercetin (Figure 1), particularly the presence and location of the hydroxyl substitutions (–OH) and the catechol-type B ring [7].

More recently, it has been proposed that the mechanisms of action of polyphenols go beyond their antioxidant activities and quercetin may also exert modulatory effects on cell signaling pathways. For instance, quercetin has anti-inflammatory effects associated to the inhibition of NF-kB [17], prevents oxidant-induced liver damage by activating the mitogen-activated protein kinases (MAPK) JNK and p38, the phosphatidylinositol-3-kinase (PI3K)/Akt pathway and the Nrf2 transcription factor [18], and induces p53-dependent apoptotic cell death in HT-29 colon cancer cells by activating AMPK [19]. Quercetin binds directly to components of the ERK MAPK pathway, namely MEK1 (MAPK Kinase Kinase) and Raf1 (MAPK Kinase) [17], and to the ATP-binding site of PI3K [20]. In *C. elegans*, it was shown that quercetin decreases ROS levels, increases the mean lifespan and attenuates the accumulation of the aging marker lipofuscin. These beneficial effects are correlated with changes in the expression of the phase II metabolism enzyme GST-4 (glutathione S-transferase) and translocation of the DAF-16 transcription factor into the nucleus [21].

Signal transduction pathways are highly conserved during evolution [22] and the yeast *S. cerevisiae* has been extensively used as an eukaryotic model organism to characterize redox cell signaling and to assess *in vivo* the antioxidant potential of natural compounds [23]–[26]. Other studies using yeast have shown that quercetin inhibits chitin synthase II [27], the H^+^-translocating Mg^2+^-ATPase from the vacuole [28] and type-2 casein kinase, Yck2p [29], a palmitoylated plasma membrane-bound serine-threonine protein kinase that is activated by Snf3p/Rtg2p glucose sensors [30]. Quercetin also prevents the nuclear localization of the Yap1p transcription factor under oxidative stress conditions [31] and induces Oye2p and Oye3p, which are involved in the modulation of actin polymerization, oxidative stress response and cell death [32]. We have previously shown an increase in H_2_O_2_ stress resistance and chronological lifespan in yeast cells treated with quercetin [33]. In this study, we have used DNA microarrays to characterize changes in the transcriptome induced by quercetin in yeast. The effect of quercetin on carbohydrate metabolism and cell wall integrity (CWI) pathway was assessed as well as its importance for oxidative stress resistance.

## Results

### Microarray Analysis of Quercetin Treated Yeast Cells

**Figure 2:**
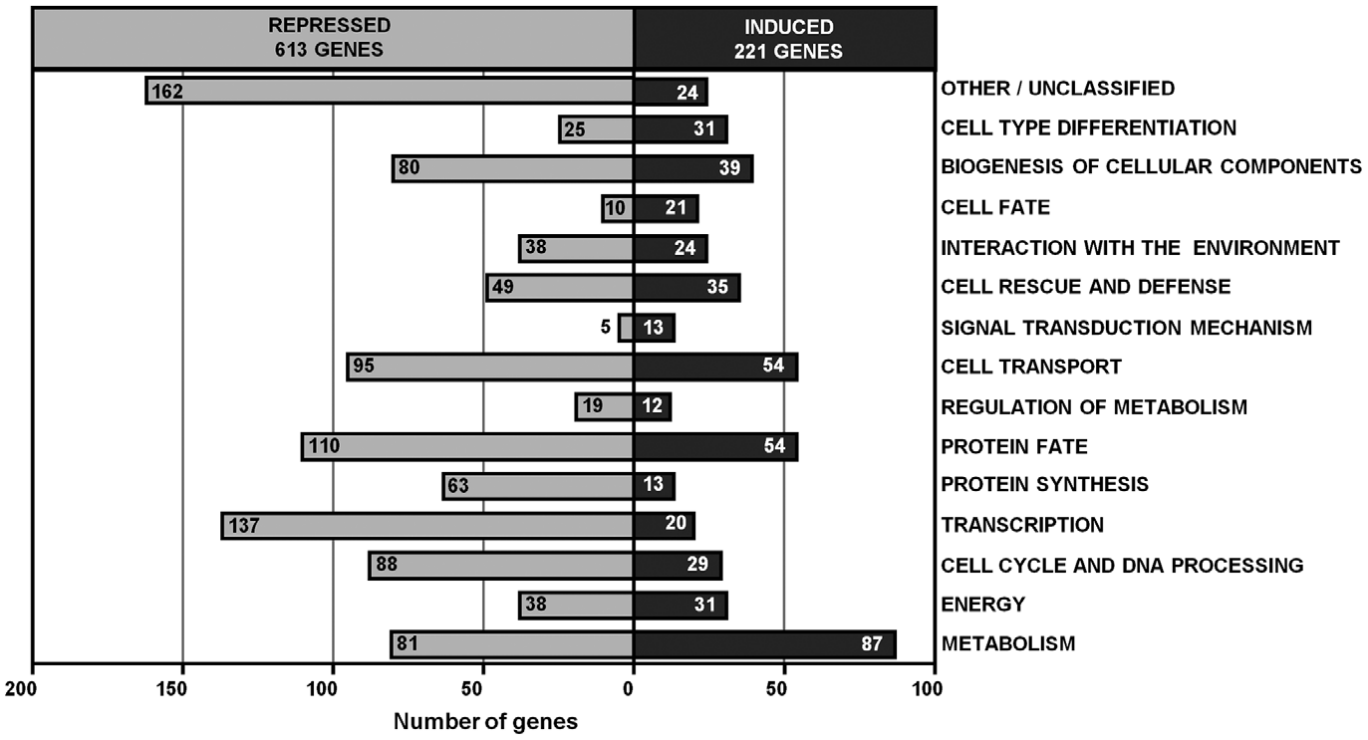
Functional categories of genes differentially expressed in quercetin-treated cells. *S. cerevisiae* BY4741 cells were grown to exponential phase and RNA was isolated as described in *Materials and Methods*. Genes up and down regulated in quercetin-treated cells were sorted in groups according to Munich Information Centre for Proteins Sequences (MIPS) database.

In a previous study, the analysis of cellular protection against oxidative stress in yeast exposed to quercetin for different time periods showed that a 15 min pre-treatment was sufficient to increase hydrogen peroxide resistance [33]. Aiming to characterize short-term adaptive responses triggered by quercetin and to identify cellular functions that may contribute to its protective effect against oxidative stress, changes in gene expression were analyzed by using microarrays. *S. cerevisiae* cells were treated with 300 µM quercetin or dimethyl sulphoxide (DMSO; control cells) during 15 min and mRNA was isolated by the hot phenol method, as described in Material and Methods. The microarray analysis showed that treatment with quercetin led to an increase of the mRNA levels of 221 genes, whereas that of 613 genes was diminished (see supplementary Tables S1 and S2). Genes differentially expressed were sorted into functional categories according to MIPS (Munich Information Center for Protein Sequences). Genes associated with transcription (22%), protein fate (18%), cell transport (15%), cell cycle and DNA processing (14%), metabolism (13%), biogenesis of cellular components (13%) and protein synthesis (10%) were down regulated, whereas genes related with metabolism (39%), protein fate (24%), cell transport (24%), biogenesis of cellular components (18%) and cell rescue and defense (16%) were up regulated by quercetin treatment (Figure 2).

**Figure 3:**
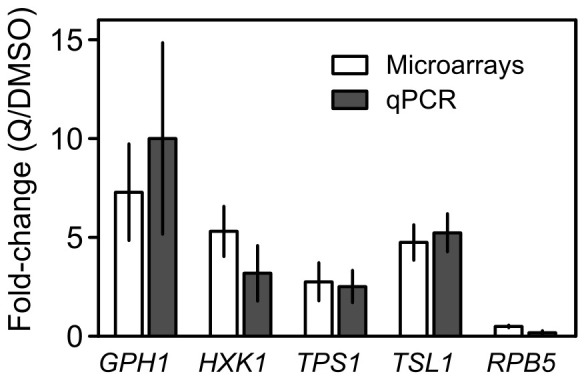
Effect of quercetin (Q) on *GPH1, HXK1, RPB5, TPS1 and TSL1* mRNA levels. Transcription profile of ***GPH1*** (glycogen phosphorylase), ***HXK1*** *(*hexokinase isoenzyme 1), ***RPB5*** (RNA polymerase subunit), ***TPS1*** (synthase subunit of trehalose-6-phosphate synthase/phosphatase complex) ***and TSL1*** (subunit of trehalose 6-phosphate synthase/phosphatase complex) **were evaluated by microarrays analysis (white bars; mean** ± SD of six arrays) and by qPCR (grey bars). The mean normalized fold expression by qPCR was calculated relative to the transcription of the reference genes *RPS6A* (component of the small (40S) ribosomal subunit) and *ACT1* (actin). The results (mean and SD of triplicates) of a representative experiment (out of three independent experiments) are shown.

To validate the microarray data, the expression of five genes was analyzed by reverse transcription quantitative polymerase chain reaction (RT-qPCR). Four of these genes were up regulated in the microarray analysis and are functionally related with the metabolism of carbohydrates, including glycogen and trehalose (whose levels were also measured; see below): *HXK1* (Hexokinase isoenzyme 1, which catalyses phosphorylation of glucose; its expression is highest during growth on non-glucose carbon sources), *GPH1* (glycogen phosphorylase), *TPS1* (synthase subunit of trehalose-6-phosphate synthase/phosphatase complex, which synthesizes trehalose) and *TSL1* (subunit of trehalose 6-phosphate synthase/phosphatase complex). The expression of *RPB5* (RNA polymerase subunit), which was down regulated in the microarray analysis, was also assessed by RT-qPCR. The mRNA levels were normalized to *ACT1* (encodes for actin; internal control) and *RPS6A* (a second control that was chosen based on the fact that its expression was not altered by quercetin, according to the microarray data). The results obtained showed a correlation between microarray and RT-qPCR data (Figure 3).

To identify biological processes overrepresented, gene lists were analyzed by using the FUNSPEC software [34]. The down regulated genes were mostly related with various aspects of RNA metabolism and ribosome biogenesis (Table 1). Notably, the expression of 14 genes containing a LSM (Like Sm) domain was repressed. LSM proteins form part of specific small nuclear ribonucleoproteins and are thought to be important modulators of RNA biogenesis and function [35]. The most important cellular functions induced include those associated with C-compound and carbohydrate metabolism, including metabolism of energy reserves, endocytosis and vacuolar proteolysis (Table 1).

**Table 1:** Functional categories overrepresented in the microarray data.

Category	p-value	In Category from Cluster	
**Down-regulated genes**			
**MIPS Functional Classification**			
rRNA processing	1.9E^−7^	40 GENES	
**MIPS Subcellular Localization**			
nucleolus	3.5E^−6^	50 GENES	
**MIPS Protein Complexes**			
RNA polymerase II	8.7E^−6^	*RPB4, RPB5, RPB8, RPB9, RPB10, RPB11, RPC10, RPO26*	
**PFam-A Domains**			
LSM	1.1E^−12^	*LSM2, LSM3, LSM4, LSM5, LSM6, LSM7, LSM8, MAK31, SMB1, SMD1, SMD2, SMD3, SME1, SMX2*	
***Up-regulated genes***			
**MIPS Functional Classification**			
Glycolysis and gluconeogenesis	8.3E^−7^		*CDC19, ENO1, GLK1, LAT1, PDA1, PGM2, PYC1, PYC2, PYK2, ZWF1*
Metabolism of energy reserves (e.g. glycogen, trehalose)	2.4E^−6^		*FKS1, GDB1, GPH1, GSY2, KRE5, NTH1, PGM2, TPS1, TPS2, TPS3, TSL1*
**MIPS Phenotypes**			
Starvation sensitivity	1.9E^−5^		*BCY1, IRA2, KEM1, PEP4, RVS167, SRV2, VRP1*
**MIPS Subcellular Localization**			
Transport vesicles	8.8E^−5^		*APM1, COP1, RPG1, SEC7, SEC16, SEC18, SEC21, SEC24*
Plasma membrane	1.9E^−4^		*CSF1, CYR1, DNF1, FKS1, FUI1, GAP1, HXT2, IRA1, IRA2, IST2, KIN1, RSP5, SEC3, SED1, SSO2, YPS1, YPS3*
**GO Biological Process**			
Vacuolar protein catabolic process	2.6E^−8^		*AIM3, ATG2, APE3, CAR2, CSF1, CWP1, GAP1, HPF1, IRA1, IST2, LAT1, NCR1, PDA1, PEP4, PRB1, PYC1, VPS13, VRP1, YIL169C*
Endocytosis	2.7E^−7^		*CHC1, DNF1, EDE1, FKS1, INP53, MYO5, NEO1, PAN1, PIL1, RSP5, RVS167, SDS24, SLA1, SVL3SWH1, VRP1*
**GO Cellular Component**			
Actin cortical patch	3.9E^−6^		*ABP1, BBC1, EDE1, FKS1, INP53, MYO5, PAN1, RVS167, SRV2, VRP1,*

### Quercetin Induces Glycogen Degradation and Trehalose Biosynthesis

Quercetin induced several genes related with carbohydrate metabolism that are functionally associated with the glycolytic/gluconeogenesis pathway. Most of these genes (*HXK1*, *GLK1*, *PGM2*, *ENO1*, *PYK2*, *PYC1* and *PYC2*) are under catabolite repression and their expression increases in response to glucose depletion [36]–[41]. Under these conditions, they function in the gluconeogenesis pathway that is activated for glucose production. Consistent with quercetin inducing a glucose restriction-like phenotype, the *HXT2* gene, which encodes a high-affinity glucose transporter induced by low levels of glucose [42], and genes that encode for the Ira1p and Ira2p GTPase-activating proteins (Table 1) were up regulated. These proteins negatively regulate RAS by converting it from the GTP- to the GDP-bound inactive form, which is required for reducing cAMP levels under nutrient limiting conditions, preventing the activation of the glucose-regulated cAMP-dependent protein kinase signaling pathway [43].

**Figure 4:**
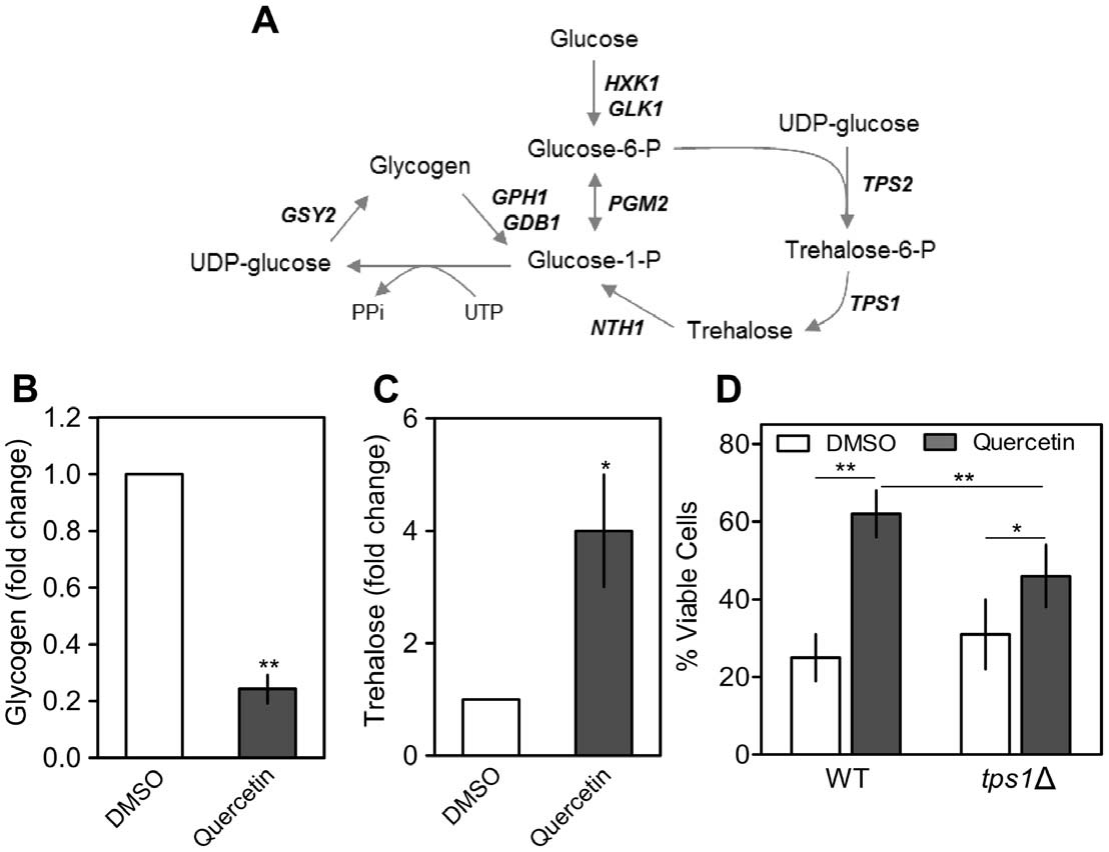
Quercetin causes glycogen depletion and increases trehalose levels. (A) Schematic representation of genes up regulated in cells treated with quercetin related with glycogen and trehalose metabolism. (B, C) *S. cerevisiae* BY4741 cells were grown to exponential phase and treated with 300 µM of quercetin or equal volume of DMSO (vehicle) for 15 min. (B) Glycogen and (C) trehalose levels were measured as described in *Materials and Methods*. Values are mean ± SD of three independent assays. *p<0.05; **p<0.01 (paired t-test). D) Hydrogen peroxide resistance. *S. cerevisiae* BY4741 (WT) and *tps1*Δ cells were treated with 300 µM of quercetin or equal volume of DMSO (control cells) for 15 min and subsequently exposed to 1.5 mM H_2_O_2_ for 1 h. Cell viability was determined by standard dilution plate counts and expressed as the percentage of the colony-forming units of non-stressed cells. Values are mean ± SD of at least three independent assays. **p<0.01; *p<0.05 (two-way ANOVA).

Moreover, quercetin increased the expression of the genes related to metabolism of energy reserves, such as glycogen (*GSY2*, *GPH1* and *GDB1*) and trehalose (*TPS1*, *TPS2*, *TSL1*, *TPS3* and *NTH1*) (Figure 4A). *GSY2* encodes the majority of the glycogen synthase activity in yeast and its expression is induced by environmental stresses, nitrogen starvation and glucose deprivation [44], [45]. *GPH1* and *GDB1* encode for glycogen phosphorylase and debranching enzyme, respectively, which are involved in glycogen degradation to glucose-1-phosphate, and their expression is induced during late exponential growth phase and in response to various stresses [46]–[48].

Trehalose is a disaccharide of glucose involved in responses to stress, including oxidative stress, as well as suppression of denatured protein aggregation [49]. Trehalose-6-phosphate synthase (Tps1p) and trehalose-6-phosphate phosphatase (Tps2p) are part of a complex containing also the regulatory proteins Tps3p and Tsl1p, which are co-induced under stress conditions and co-repressed by the glucose regulated Ras-cAMP pathway [50]. Notably, *NTH1* gene that encodes a neutral trehalase was also up regulated in cell treated with quercetin. *NTH1* is a multiple stress response gene [51]. The simultaneous increase of trehalose hydrolyzing and synthesizing enzymes also occurs during heat stress. This seemingly futile cycling of trehalose turnover during heat stress seems to be necessary for maintenance of a constant glucose concentration in the cytosol and for cellular recovery from stress [52], [53].

Changes in expression of genes related to glycogen and trehalose metabolism led us to measure the levels of these reserve carbohydrates. The results obtained show that glycogen levels decreased approximately 4-fold whereas trehalose levels increased 4-fold upon quercetin-treatment (Figure 4B–C). Overall, the gene expression results and the carbohydrate levels suggest that glycogen is metabolized to provide glucose for trehalose biosynthesis. Moreover, the protective effect of quercetin against H_2_O_2_ decreased in *tps1*Δ cells (Figure 4D): in the presence of this polyphenol, oxidative stress resistance increased 2.5-fold and 1.5-fold in wild type and *tps1*Δ cells, respectively. This suggests that trehalose production contributes to quercetin-induced H_2_O_2_ resistance.

### Quercetin Activates the Cell Wall Integrity Pathway

**Figure 5:**
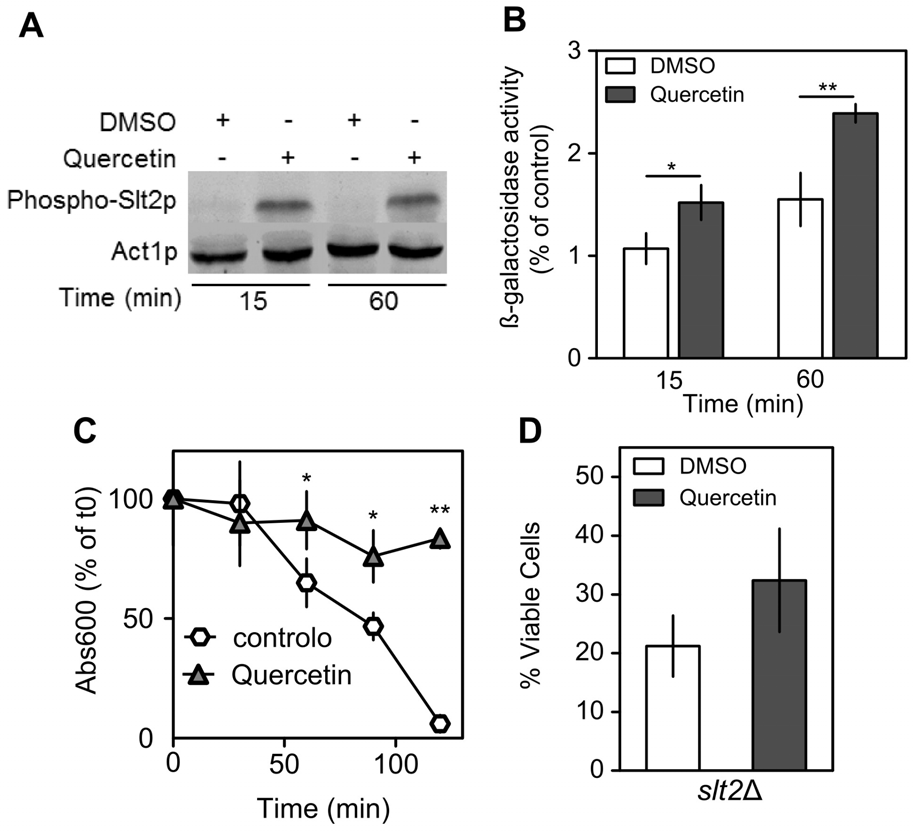
Quercetin activates the cell wall integrity signaling pathway. (A) Phospho-Slt2 protein levels. *S. cerevisiae* BY4741 cells were grown to exponential phase and treated with 300 µM of quercetin or equal volume of DMSO for the indicated time periods. Proteins were isolated, separated by SDS-PAGE, blotted into a membrane and incubated with anti-phospho-p44/42 antibody that detects dually phosphorylated Slt2p or with anti-actin antibody (loading control), as described in *Materials and Methods*. A representative blot is shown. (B) Rlm1p-transcription factor activity. Exponentially growing *S. cerevisiae* BY4741 cells transformed with a *pRLM1-LacZ* reporter construct were treated with 300 µM of quercetin or equal volume of DMSO (control) at the indicated times. β-galactosidase activity was determined as described in *Materials and Methods* and expressed as percentage of control cells. (C) Resistance to zymolyase digestion. *S. cerevisiae* BY4741 cells were treated with 300 µM of quercetin or equal volume of DMSO for 15 min and incubated with 0.25 U/ml zymolyase at 37°C. Cell lysis was determined spectrophotometrically at 600 nm over time. (D) Hydrogen peroxide resistance of *slt2*Δ cells was assessed as described in legend to Figure 4D. Values are mean ± SD of at least three independent assays. **p<0.01; *p<0.05 (unpaired t-test).

Quercetin also increased the expression of genes encoding proteins that exhibit a cortical patch membrane localization pattern (Table 1), which are involved in the regulation of actin cytoskeleton, endocytosis, cell wall biogenesis and viability following starvation or osmotic stress [54], [55]. Moreover, quercetin up regulated several genes that encode proteins related to the cell wall, such as Sed1p (cell wall glycoprotein), Yps1p and Yps3p (aspartic proteases), and Fks1p (1,3- β-D-glucan synthase) (Table 1). Cell wall biogenesis and maintenance are regulated by the CWI pathway, a Pkc1p-modulated MAPK cascade [56]. The MAPK module is composed of the MAPK kinase kinase Bck1p, a pair of redundant MAPK kinases (Mkk1p and Mkk2p), and the MAPK Slt2p/Mpk1p. To test if quercetin activates this pathway, phospho-Slt2p levels were analyzed by Western blotting, using an anti-phospho-p44/42 antibody that detects dually phosphorylated Slt2p, the active form of this MAPK. Phospho-Slt2p levels were significantly increased in cells treated with quercetin for 15 and 60 min (Figure 5A). We also characterized changes in Rlm1p, a transcription factor regulated by Slt2p [57], by measuring β-galactosidase activity in cells expressing a LacZ reporter under the control of Rlm1p (*RLM1*-*lacZ*). Consistent with an induction of the Rlm1p-reporter, β-galactosidase activity increased 50% in cells treated with quercetin (Figure 5B). The activation of the CWI pathway is important for oxidative stress resistance [58] and increases cellular resistance to cell wall perturbing agents, such as zymolyase, a lytic enzyme that degrades β1,3-glucans, the main component of cell wall. Consistent with Slt2p activation, quercetin increased zymolyase resistance (Figure 5C). Moreover, inactivation of *SLT2* gene decreased the protective effect of quercetin against H_2_O_2_ (Figure 5D). These results indicate that quercetin increases H_2_O_2_ resistance through activation of the CWI pathway.

### Quercetin does not Prevent H_2_O_2_-induced Actin Polarization

**Figure 6:**
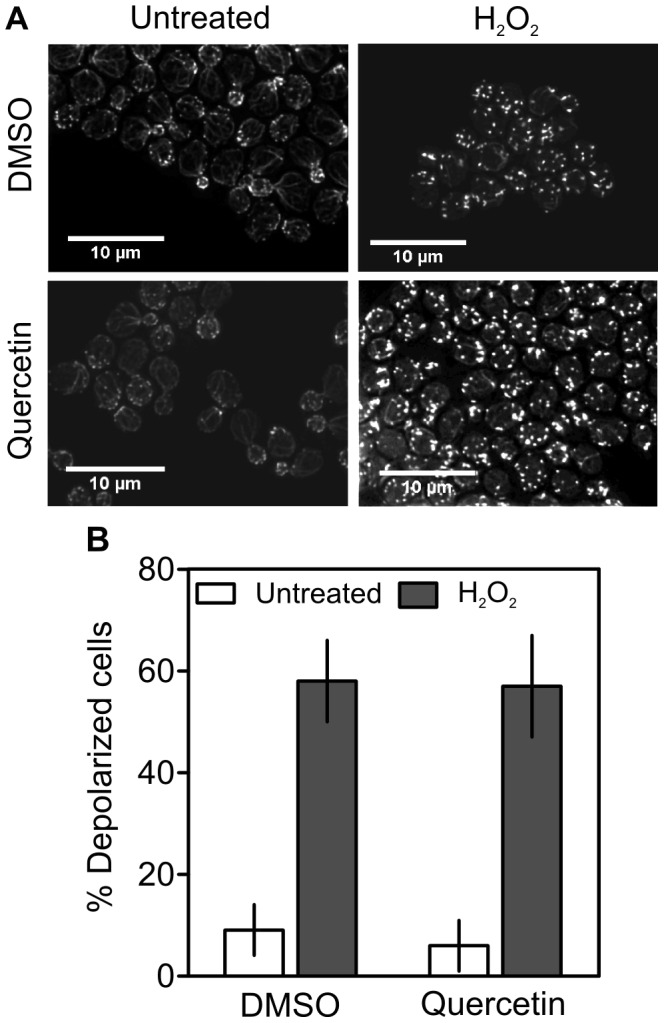
Quercetin does not affect H_2_O_2_-induced actin depolarization. Yeast cells were grown to exponential phase and pre-treated with 300 µM of quercetin or equal volume of DMSO (control cells) for 15 min and subsequently exposed to 1.5 mM H_2_O_2_ for 1 h. Cells were incubated with rhodamine-phalloidin to stain actin and visualized on a fluorescence microscope. (A) Deconvolved and stack fluorescence images of cells with actin staining; (B) Percentage of depolarized cells obtained after counting approximately 1500 cells for each treatment condition. Data represent the mean ± SD of three independent assays.

Actin has also been implicated in oxidative stress resistance. Oxidation of yeast actin decreases its dynamics, causing depolarization of the mitochondrial membrane and an increase in ROS production that contributes to cell death [59]. To test if quercetin affects actin dynamics, cells were pre-treated with DMSO or quercetin for 15 min and exposed to 1.5 mM H_2_O_2_ during 1 h. To visualize actin, cells were labeled with the rhodamine-phalloidin, a high-affinity F-actin probe conjugated to the red-orange fluorescent dye, tetramethylrhodamine, and observed by fluorescence microscopy. In control cells (pre-treated with DMSO), almost 60% of the cells displayed actin depolarization after exposure to H_2_O_2_ (Figure 6). Similar results were observed in cells pre-treated with quercetin, suggesting that its protective effect is not associated with changes in actin polarization.

## Discussion

Oxidative stress has been implicated in aging and age-related diseases. Mounting evidence suggests that natural compounds with antioxidant properties exert health beneficial effects. The antioxidant activities of polyphenols have been attributed to ROS scavenging. However, a number of recent studies suggest that they also act through modulation of cell signaling pathways that increase cellular defense mechanisms. This phenomenon, known as xenohormesis, refers to sensing in one organism (yeast in this study) of a compound produced in another specie (plant) in response to environmental stress, leading to the induction of a defense response that increases its chances of survival [60]. We have previously shown that quercetin, the most common flavonol in the diet, increases oxidative stress resistance in yeast cells by scavenging free radicals, maintaining the redox homeostasis, and preventing protein carbonylation and lipid peroxidation [33]. Aiming to characterize genome-wide changes in gene expression induced by quercetin in yeast, a microarray analysis was performed. The results obtained show that quercetin down regulated a significant number of genes belonging to RNA metabolism and ribosome biogenesis categories. This cellular adaptation has been observed in response to multiple stress conditions [61] and is beneficial since it spares energy resources for cellular processes other than ribosome biosynthesis (the most energy consuming cellular process).

In yeast, the acquisition of oxidative stress resistance has been associated with adaptive responses that include the induction of antioxidant defences and other stress proteins, mediated by Yap1p, Skn7p and Msn2p transcription factors [62], [63]. The analysis of the promoters of the genes differentially expressed upon quercetin treatment, using YEASTRACT software [64], showed that quercetin did not affect the expression of genes known to be regulated by Yap1p or Msn2p, suggesting that these factors are not key mediators of quercetin protective effects (see supplementary Tables S3 and S4). Although a few Skn7p-target genes were up regulated, they represent 5.2% of the total number of genes directly regulated by this transcription factor. Thus, it is likely that changes in gene expression induced by quercetin are Skn7p-independent.

Notably, quercetin induced several genes belonging to carbohydrate metabolism, including metabolism of energy reserves, known to be repressed by glucose. Specifically, quercetin up regulated genes associated with gluconeogenesis, glycogenolysis, glucose uptake and trehalose biosynthesis, as well as *IRA1* and *IRA2* genes, which encode GTPase-activating proteins that negatively regulate the glucose-activated cAMP-dependent protein kinase signaling pathway [43]. These metabolic alterations redirect carbohydrate metabolism towards the production of trehalose, whose levels increased 4-fold after exposure to quercetin. Trehalose-6-phosphate, one of the intermediates of this pathway, and Tps1p play a major role in restricting glucose influx into glycolysis [65]. Trehalose is a disaccharide of glucose with stress-protectant functions [49]. Our results indicate that the increase in trehalose production contributes to oxidative stress resistance of cells treated with quercetin, since inactivation of *TPS1* gene decreased its protective effect. Interestingly, glycogen levels also decrease whereas trehalose levels remain unchanged in stationary phase (quiescent) cells, which display a high oxidative stress resistance phenotype. Under these conditions, trehalose also functions as an energy reserve used by yeast cells upon exit from the quiescent state [66].

This adaptive, xenohormetic response to quercetin includes features observed under glucose derepression conditions, suggesting that quercetin induces glucose restriction-like phenotypes. Some of these changes were also described for mammalian cells. Quercetin has been shown to stimulate glycogenolysis [67] and to inhibit glucose uptake, insulin signaling and activation of Akt, a downstream effector of PI3K [68], [69]. Quercetin and other flavonoids may also compete with glucose for transmembrane transport [68].

Genes encoding proteins that exhibit a cortical patch membrane localization pattern were also up regulated by exposure to quercetin. These proteins are involved in the regulation of actin cytoskeleton and cellular processes such as cell wall biogenesis [54], [55]. Changes in actin polarization increases mitochondrial ROS production [59]. However, quercetin did not decrease actin depolarization induced by H_2_O_2_, suggesting that its protective effect is not associated with the modulation of actin dynamics. In contrast, our results suggest that quercetin exerts its protective effects through the activation of cell wall biogenesis and maintenance. Indeed, this flavonol up regulated several genes encoding cell wall proteins and activated components of the CWI pathway, namely the Slt2p MAPK and the Rlm1p transcription factor [57]. In addition, the increase of H_2_O_2_ resistance induced by quercetin was attenuated in *slt2*Δ mutant cells. These results support a role for CWI pathway in oxidative stress resistance, as previously suggested [58]. Consistent with CWI pathway activation, quercetin also increased cell resistance to zymolyase, a lytic enzyme that causes cell wall stress by degrading β1,3-glucans, the main component of cell wall. The modulation of signal transduction mediated by PKC was also observed in colon cancer cell lines exposed to quercetin [70].

In conclusion, this study shows that quercetin treatment mimics glucose restriction and modulates carbohydrate metabolism in favor of the biosynthesis of trehalose, a disaccharide with antioxidant properties. Moreover, the activation of the CWI pathway in yeast contributes to the xenohormetic activity of quercetin. The overall results suggest that quercetin exerts protective effects through the modulation of cell signaling pathways.

## Materials and Methods

### Reagents

All reagents and chemicals were of analytical grade. Sodium or potassium phosphates and H_2_O_2_ were purchased from Merck (VWR, Lisboa, Portugal), yeast nitrogen base from Difco (Quilaban, Sintra, Portugal), agarose from Invitrogen (Alfagene, Carcavelos, Portugal), and DMSO, quercetin and zymolyase from Sigma (Sintra, Portugal). Quercetin was dissolved in DMSO at a 200 mM stock concentration and stored at −80°C. Solutions were prepared in ultrapure water (MilliQ).

### Yeast Cells and Growth Conditions

The *Saccharomyces cerevisiae* cells (Euroscarf, Germany) used in this study were BY4741 (Matα, *his3*Δ_1_, *leu2*Δ_0_, *met15*Δ_0_, *ura3*Δ_0_), *slt2*Δ (BY4741 *slt2*Δ::*KanMX4*) and *tps1*Δ (BY4741 *tps1*Δ::*KanMX4*). Yeast cells were grown in minimal medium [0.67% (w/v) yeast nitrogen base with ammonium sulfate without amino acids and 2% (w/v) glucose] supplemented with appropriate amino acids (40 mg His L^−1^, 80 mg Leu L^−1^, 40 mg Met L^−1^) and nucleotides (40 mg Ura L^−1^). Auxotrophic supplementation was performed before autoclaving at 110°C. Cells were grown to early exponential phase (Abs_600nm_  =  0.6) in an orbital shaker, at 26°C and 120 rpm, with a ratio of flask volume / medium volume of 5∶1. To test the activity of the Rlm1p transcription factor, *S. cerevisiae* BY4741 cells were transformed by electroporation with p2xRlm1-LacZ plasmid [71] and selected in minimal medium lacking uracil.

### RNA Extraction and mRNA Isolation

Exponential phase cells (90 Abs_600nm_ units of yeast) were exposed to 300 µM quercetin (dissolved in DMSO) or equal volume of DMSO (control) by directly adding these compounds to the growth media. After 15 min of treatment, cells were collected by centrifugation at 2,900 g. Total RNA was extracted by a hot acidic phenol method [72], with a yield of approximately 1500 µg. Briefly, cells were resuspended in 500 µL acid phenol:chloroform 5∶1 pH 4.7 and the same volume of TES buffer [10 mM Tris pH 7.5 10 mM ethylenediaminetetraacetic acid (EDTA) 0.5% sodium dodecyl sulfate (SDS)], and incubated at 65°C for 1 h, with occasional, brief vortexing. After centrifugation at 10,000 g for 20 min, RNA was isolated from the aqueous phase by extractions with phenol:chloroform 5∶1 pH 4.7 (twice) and chloroform:isoamyl alcohol 25∶1, and precipitation with 100% ethanol, in the presence of sodium acetate pH 5.2. RNA was dissolved in water and its purity assessed by gel electrophoresis in 1.4% (w/v) agarose and by UV spectrophotometry, using NanoDrop ND-1000 Spectrophotometer. Only RNA samples with A_260/280_ ratio >1.90 were used in further experiments. For microarray studies, mRNA was isolated from 1500 µg of total RNA, as described in Oligotex^TM^ Handbook (Qiagen^®^), with a yield of approximately 6 µg. For hybridization quality control, mixtures of ten *in vitro* synthesized RNAs were added from appropriately diluted mixtures to the total RNA samples prior to mRNA enrichment.

### cDNA Synthesis and Labeling

cDNA synthesis and labeling were performed as described previously [73]. Briefly, cDNA synthesis was carried out from 3 µg of mRNA enriched samples in the presence of 2-aminoallyl-dUTP (Sigma). cDNA was purified using Microcon YM-30 columns (Millipore, VWR, Lisboa, Portugal) prior to coupling to NHS ester Cy3 or Cy5 (GE Healthcare). Uncoupled dye was removed using Chromaspin-30 columns (Clontech, Enzifarma S.A., Oeiras, Portugal). The efficiency of cDNA synthesis and of dye incorporation was measured by spectrophotometry (NanoDrop). Samples with a degree of labeling (labeled nucleotides per 100 nucleotides) outside the range of 5.6 ± 1.3 were not considered for hybridization.

### Microarray Hybridization

Hybridizations were carried out as previously described [73]. Samples derived from quercetin treated cells were labeled with Cy5 and were co-hybridized with control (DMSO) samples labeled with Cy3. Dye-swap hybridization replicates were performed to reduce dye-specific biases in signal intensity. For each comparison (treatment vs. control), 3 biological replicates were analyzed in 2 dye-swapped hybridizations each, in a total of 6 hybridizations. The same amount of differentially labeled treatment and control cDNA (0.30 µg) was mixed and hybridized with microarrays containing 6388 oligonucleotide probes representing the yeast ORFeome in duplicate, fabricated in the laboratory of RNA biology at University of Aveiro, Portugal. A set of 70 mer probes used to detect the spiked-in control RNA added to the total RNA sample were also included in the microarray design, in order to monitor labeling and hybridization quality. Details of the microarray composition can be found in ArrayExpress public database (http://www.ebi.ac.uk/microarray-as/ae/) under the accession number A-MEXP-1185.

### Image Acquisition and Data Analysis

Microarray images were obtained with the Agilent G2565BA microarray scanner. Raw data was extracted using QuantArray v3.0 software (PerkinElmer). Pre-processing and normalization of the data was performed using the Biometric Research Branch (BRB)-ArrayTools v3.4.0 software. Briefly, manually flagged bad spots were eliminated and the local background was subtracted prior to averaging of replicate features on the array. Log2 intensity ratios (M values) were adjusted by global Lowess normalization. The MIAME (Minimum Information About a Microarray Experiment) compliant data was deposited in ArrayExpress (http://www.ebi.ac.uk/miamexpress/) under the accession number E-MEXP-3523.

Treatment and control samples were compared using Significance Analysis for Microarrays (SAM) [74] implemented in TM4 Microarray Software Suite (MeV) 4.3 [75]. Significance analysis, with a false discovery rate (90^th^ percentile) of 0.016%, resulted in a common set of 1687 significant genes, from which 834 were selected based on an average fold change of more than 2.0. Statistical analysis of overrepresentation of functional groups was performed by using FUNSPEC [34]. All available databases were addressed by using probability cut-off of 10^−3^ and the Bonferroni correction for multiple testing. Genes differentially expressed in quercetin-treated cells were sorted in functional groups according to Munich Information Centre for Protein Sequences (MIPS) database (Tables S1 and S2).

### Reverse Transcription Quantitative Polymerase Chain Reaction (RT-qPCR)

Glycogen phosphorylase (*GPH1*), hexokinase I (*HXK1*), RNA polymerase subunit B (*RPB5*), trehalose-6-phosphate synthase (*TPS1*) and trehalose synthase subunit (*TSL1*) transcription profile was validated by RT-qPCR. For each analysis *RPS6A* and *ACT1* were used as internal normalizers. Two micrograms of DNase I-treated (Promega, VWR, Lisboa, Portugal) total RNA were transcribed with the iScriptTM Select cDNA Synthesis Kit (Bio-Rad, Amadora, Portugal), using the random primers supplied and following the manufacturer’s recommendations. Control PCRs were performed with RT-untreated RNA to confirm the absence of contaminating DNA in the RNA preparations. qPCR amplifications were performed using 2 µL of template cDNA, 10 µL of SYBR Green Supermix (Bio-Rad) and 0.2 µM of each primer (Table 2). Reactions were carried out in the iCycler iQ5 Real-Time PCR detection system (Bio-Rad). Cycling conditions were as follows: 95°C for 3 min; 40 cycles of 95°C for 30 s, 56°C or 57°C (according to the set of primers used) for 60 s and 72°C for 30 s. Standard dilutions of the cDNA were used to check the relative efficiency and quality of the primers. Negative controls (no template cDNA) were included in all qPCR. A melting curve analysis was performed at the end of each qPCR assay to exclude the formation of non-specific products. The obtained data was analyzed using the software provided by the supplier (iQ5 Optical System Software v2.1, Bio-Rad) and relative expression of the selected genes in quercetin-treatment vs. control cells was determined using the Pfaffl method [76].

**Table 2:** Primers used in real-time PCR.

Primer^a^	Systematic Name	Sequence
ACT1_S	YFL039C	TGGATTCTGAGGTTGCTGCTTT
ACT1_AS	YFL039C	CCGACGATAGATGGGAAGACAG
RPS6A_S	YPL090C	TCGGTCAAGAAGTCGATGGTGA
RPS6A_AS	YPL090C	CACCTTGCTTCATTGGGAAACC
GPH1_S	YPR160W	ACATGGCTGCTTATGAAGCTGC
GPH1_AS	YPR160W	TCCAAAGCCCTACCCATCAAA
HXK1_S	YFR053C	AGGTCCAAAGAAACCACAGGCT
HXK1_AS	YFR053C	AACCGGGAATCATTGGAATGTT
RPB5_S	YBR154C	TCAAGAGGAAGTCGAATTGCCA
RPB5_AS	YBR154C	TGGATTTGCCTGGAAGGACA
TPS1_S	YBR126C	CGATGAGAAGGATCAGGTGAGG
TPS1_AS	YBR126C	GATGGTAATGGAATAACGGCCA
TSL1_S	YML100W	CGCCAATCCAACAGCAACAG
TSL1_AS	YML100W	GAAGTAGCCGCCGAAGTAGG

^a^S, sense; AS, antisense.

### Trehalose and Glycogen Assays

The content of intracellular trehalose and glycogen was determined in cells (30 Abs_600nm_ units of yeast) treated with quercetin or DMSO, as described above, by measuring glucose released by trehalase or amyloglucosidase, respectively [77]. The amount of glucose released from trehalose and glycogen was determined using glucose (GO) assay kit (Sigma), according to manufacturer’s recommendations. Results are expressed as fold change relative to control (DMSO-treated) cells.

### Oxidative Stress Resistance

Yeast cells (2×10^7^/mL) were pre-treated with quercetin or DMSO for 15 min, as described above, and subsequently exposed to 1.5 mM H_2_O_2_ for 1 h. Cell viability was determined by standard dilution plate counts on YPD medium containing 1.5% (w/v) agar. Colonies were counted after growth at 26°C for 3 days. Viability was expressed as the percentage of the colony-forming units.

### Actin Staining with Rhodamine-phalloidin

Yeast cells (3 Abs_600nm_ units) were pre-treated with quercetin or DMSO for 15 min and subsequently exposed to 1.5 mM H_2_O_2_ for 1 h, as described above. Cells were then fixed by adding directly to the culture 3% formaldehyde for 10 min, washed twice with phosphate-buffered saline (PBS) and fixed in 10% formaldehyde/PBS for 1 h with agitation at room temperature. Cells were washed twice in PBS and permeabilized in 1% tritonX100/PBS for 3 min and washed again with PBS. For actin stain, cells were incubated in the dark for 1 h with 200 U/ml rhodamine-phalloidin/PBS (Invitrogen) and washed one time with PBS before resuspending in 20 µl of anti-bleaching solution (Vectashield, Vector Lab. Inc.) and mounted on agarose beds. All centrifugations were performed at 1,600 g 5 min. Z-stacks images were acquired using a AxioImager Z1^®^ fluorescence microscope (Axiovision 4.7^®^ acquisition software) equipped with a 43HE filter set for rhodamine-phalloidin, a 40x oil-immersion objective and an Axiocam MR ver.3.0^®^ (Carl Zeiss). Z-stack images were deconvolved using Huygens^®^ Essential software. Image stacks and cell counting were performed using Image J/Fiji^®^ software. Cells with an irregular and sparse distribution pattern of rhodamine-phalloidin fluorescence were considered depolarized.

### Western Blotting

Yeast cells (30 Abs_600nm_ units) were exposed to quercetin or DMSO for 15 or 60 min, as described above, and collected by centrifugation at 2,900 g. Yeast extracts were prepared in 50 mM potassium phosphate buffer (pH 7.0) containing protease inhibitors (Complete, Mini, EDTA-free Protease Cocktail Inhibitor Tablets; Roche Applied Science, Amadora, Portugal) and phosphatase inhibitors (50 mM sodium fluoride, 5 mM sodium pyrophosphate, 1 mM sodium orthovanadate), by vigorous shaking of the cell suspension in the presence of glass beads for 5 min. Short pulses of 1 min were used, with 1 min intervals on ice. Cell debris was removed by centrifugation at 10,000 g for 15 min at 4°C and protein content was determined by the Lowry method, using bovine serum albumin as a standard. Proteins (50 µg) were separated by SDS-PAGE using 10% polyacrylamide gels and blotted onto a nitrocellulose membrane (Hybond-C, GE Healthcare). The membrane was incubated with rabbit anti-phospho-p44/42 MAPK (Cell Signaling Technology, Izasa, Lisboa, Portugal) at a 1∶2,000 dilution or rabbit anti-actin (Sigma) at a 1∶200 dilution as primary antibodies to detect phospho-Slt2p and actin (loading control), respectively, as previously described [78]. Subsequently, the membrane was incubated with the secondary antibody, goat anti-rabbit IgG-peroxidase (Sigma), at a 1∶5,000 dilution. Immunodetection was performed by chemiluminescence, using a kit from GE Healthcare (RPN 2109). Quantification of band intensities was performed by densitometry using Quantity One package (Bio-Rad).

### β-galactosidase Activity

Yeast cells (30 Abs_600nm_ units) expressing the p2xRlm1-LacZ reporter or pLGΔ178 were treated with quercetin or DMSO for 15 or 60 min, as described above, and collected by centrifugation at 2,900 g. The β-galactosidase activity was measured as previously described [33].

### Cell Wall Stress Assay

For the analysis of zymolyase sensitivity, yeast cells (2×10^7^/mL) were treated with quercetin or DMSO for 15 min, as described above, washed twice, and resuspended in 10 mM Tris-HCl pH 7.5. Cells were then incubated with 0.25 U/ml zymolyase at 37°C and absorbance at 600 nm followed overtime. A decrease in Abs_600nm_ indicates cell lysis due to degradation of β1,3-glucans, the main component of cell wall.

### Statistical Analysis

Data are expressed as mean values ± standard deviation of independent experiments. Values were compared by Student’s t-test or Two-way ANOVA using GraphPad Prism Software v5.01 (GraphPad Software).

## Supporting Information

Table S1 **Genes up regulated in quercetin treated cells.**
(PDF)

Table S2 **Genes down regulated in quercetin treated cells.**
(PDF)

Table S3 **Transcription factors with documented direct regulation of genes induced by exposure to quercetin.**
(PDF)

Table S4 **Transcription factors with documented direct regulation of genes repressed by exposure to quercetin.**
(PDF)
