# Supplementary material for: Quercetin Protects Saccharomyces cerevisiae against Oxidative Stress by Inducing Trehalose Biosynthesis and the Cell Wall Integrity Pathway
Source: PLoS One. 2012 Sep 18;7(9):e45494. doi: 10.1371/journal.pone.0045494 (PMC3445532; doi:10.1371/journal.pone.0045494)
Supplement: Table S1 — Genes up regulated in quercetin treated cells. (PDF) [file pone.0045494.s001.pdf]

Table S1 Genes up regulated in quercetin treated cells. *S. cerevisiae* BY4741 was grown to exponential phase in YPD medium and treated with 300 µM of quercetin or equal volume of DMSO (control cells) for 15 min. Genes differentially regulated (quercetin *vs* control) were sorted into functional categories according to MIPS; some genes were listed in more than one category.

Gene Name	Fold change	Gene product
**Amino acid metabolism**		
"DUR1,2"	3.0	urea amidolyase (contains urea carboxylase and allophanate hydrolase)
SHM1	2.0	"Serine hydroxymethyltransferase, mitochondrial"
ILV6	2.6	"Regulatory subunit of acetolactate synthase, which catalyzes the first step of branched-chain amino acid biosynthesis; enhances activity of the Ilv2p catalytic subunit, localizes to mitochondria"
ILV1	2.0	threonine deaminase
THR1	2.1	homoserine kinase
APE2	3.4	aminopeptidase yscII
ECM38	2.2	gamma-glutamyltransferase homolog
CAR2	2.7	ornithine aminotransferase
GCV2	2.3	glycine cleavage system P subunit|glycine decarboxylase complex P subunit|glycine synthase P subunit
GAD1	2.2	glutamate decarboxylase
ZWF1	3.3	glucose-6-phosphate dehydrogenase
ARG1	2.0	arginosuccinate synthetase
GDH1	2.0	NADP-specific glutamate dehydrogenase
**Nucleotide/nucleoside/nucleobase metabolism**		
CDC19	2.6	pyruvate kinase
FUI1	2.0	uridine permease
MEC1	2.4	"Required for mitotic growth, DNA repair and mitotic recombination, regulates phosporylation of Rad53p, required for dmc1 arrest and meiotic recombination, homolog of fission yeast Rad3 and human ATR"
"DUR1,2"	3.0	urea amidolyase (contains urea carboxylase and allophanate hydrolase)
SDS24	2.1	"Similar to S. pombe SDS23, suppresses DIS2, localized to the nucleus"
SHM1	2.0	"Serine hydroxymethyltransferase, mitochondrial"
GDA1	2.5	guanosine diphosphatase of Golgi membrane
KEM1	2.2	5'-3' exonuclease
"ADE5,7"	2.1	aminoimidazole ribotide synthetase|glycinamide ribotide synthetase
CYR1	2.5	adenylate cyclase
URA2	2.5	aspartate transcarbamylase|carbamoyl phosphate synthetase|glutamine amidotransferase
JSN1	2.1	benomyl dependent tubulin mutant
DCS1	1.9	"Non-essential hydrolase involved in mRNA decapping, may function in a feedback mechanism to regulate deadenylation, contains pyrophosphatase activity andJa HIT (histidine triad)Jmotif; interacts with neutral trehalase Nth1pJJ"
ADE17	2.6	5-aminoimidazole-4-carboxamide ribonucleotide (AICAR) transformylase/IMP cyclohydrolase
SRV2	2.4	70 kDa adenylyl cyclase-associated protein
IRA2	2.3	GTPase activating protein|highly homologous to Ira1p|neurofibromin homolog
PYK2	2.6	"Pyruvate kinase, glucose-repressed isoform"
**Phosphate metabolism**		
CDC19	2.6	pyruvate kinase
SEC18	2.1	ATPase|NSF|protein involved in protein transport between ER and Golgi

Gene Name	Fold change	Gene product
MEC1	2.4	"Required for mitotic growth, DNA repair and mitotic recombination, regulates phosporylation of Rad53p, required for dmc1 arrest and meiotic recombination, homolog of fission yeast Rad3 and human ATR"
GLK1	3.0	glucokinase
TPS2	2.2	trehalose-6-phosphate phosphatase
KIN1	2.0	Serine/threonine protein kinase
PDR15	2.0	multidrug resistance transporter (putative)
GDA1	2.5	guanosine diphosphatase of Golgi membrane
GAL83	2.0	Glucose repression protein
PTC2	2.1	protein phosphatase type 2C
CHD1	2.3	transcriptional regulator
HXK1	5.2	hexokinase I (PI) (also called hexokinase A)
PIL1	2.4	Phosphorylation Inhibited by Long chain base
THR1	2.1	homoserine kinase
KSP1	2.6	Serine/threonine kinase similar to casein kinase II and other serine/threonine protein kinases
SCH9	2.1	cAMP-dependent protein kinase homolog|suppressor of cdc25ts
TPK1	2.0	cAMP-dependent protein kinase catalytic subunit (putative)
SSC1	2.4	mitochondrial matrix protein involved in protein import|Endo.SceI endonuclease subunit
HSP104	2.4	heat shock protein 104
YBT1	2.1	"Yeast bile transporter, similar to mammalian bile transporter"
DAK1	2.0	dihydroxyacetone kinase (putative)
YNR047W	2.1	questionable protein
INP53	2.0	inositol polyphosphate 5-phosphatase
PYK2	2.6	"Pyruvate kinase, glucose-repressed isoform"

C-compound and carbohydrate metabolism

CDC19	2.6	pyruvate kinase
TPS1	2.6	trehalose-6-phosphate synthase/phosphatase complex 56 kDa synthase subunit
PYC2	2.5	pyruvate carboxylase
SHM1	2.0	"Serine hydroxymethyltransferase, mitochondrial"
ILV6	2.6	"Regulatory subunit of acetolactate synthase, which catalyzes the first step of branched-chain amino acid biosynthesis; enhances activity of the Ilv2p catalytic subunit, localizes to mitochondria"
GLK1	3.0	glucokinase
SRB8	2.7	activation mediator subcomplex of RNA polymerase I holoenzyme
GPD1	2.1	glycerol-3-phosphate dehydrogenase
BSC1	4.0	Bypass of Stop Codon transcript encoded by this ORF shows a high level of stop codon bypass
NTH1	2.3	neutral trehalase
TPS2	2.2	trehalose-6-phosphate phosphatase
ADR1	2.7	positive transcriptional regulator
SSN2	2.2	transcription factor
EMI2	2.7	Early Meiotic Induction
GAL83	2.0	Glucose repression protein
HXK1	5.2	hexokinase I (PI) (also called hexokinase A)
CWH41	2.1	glucosidase I
PYC1	2.5	pyruvate carboxylase
DIE2	2.3	glucosyltransferase
ENO1	2.1	enolase I
GRE3	2.0	aldose reductase
BCY1	2.0	cAMP-dependent protein kinase regulatory subunit
KGD1	2.4	alpha-ketoglutarate dehydrogenase
SUC2	2.6	invertase (sucrose hydrolyzing enzyme)
PMT4	2.0	dolichyl phosphate-D-mannose:protein O-D-mannosyltransferase

Gene Name	Fold change	Gene product
GFA1	3.2	glucoseamine-6-phosphate synthase|glutamine_fructose-6-phosphate amidotransferase
SDH1	2.2	succinate dehydrogenase flavoprotein subunit
GPI13	2.0	ER membrane localized phosphoryltransferase that adds phosphoethanolamine onto the third mannose residue of the glycosylphosphatidylinositol (GPI) anchor precursor; similar to human PIG-O protein
PDC5	2.3	pyruvate decarboxylase
GSY2	2.2	glycogen synthase (UDP-glucose-starch glucosyltransferase)
ECM38	2.2	gamma-glutamyltransferase homolog
FKS1	2.6	"1,3-beta-D-glucan synthase"
DAK1	2.0	dihydroxyacetone kinase (putative)
TSL1	4.7	similar to TPS3 gene product|trehalose-6-phosphate synthase/phosphatase complex 123 kDa regulatory subunit
PGM2	4.1	phosphoglucomutase
ADE17	2.6	5-aminoimidazole-4-carboxamide ribonucleotide (AICAR) transformylase/IMP cyclohydrolase
GCV2	2.3	glycine cleavage system P subunit|glycine decarboxylase complex P subunit|glycine synthase P subunit
YMR237W	2.1	questionable protein
TPS3	3.1	trehalose-6-phosphate synthase/phosphatase complex 115 kDa regulatory subunit
LAT1	2.5	pyruvate dehydrogenase complex dihydrolipoamide acetyltransferase component (E2)
ZWF1	3.3	glucose-6-phosphate dehydrogenase
YOL155C	2.1	ORF
LSC1	2.0	"alpha subunit of succinyl-CoA ligase (synthetase; ATP-forming), a mitochondrial enzyme of the TCA cycle"
KRE5	2.2	"appears to function early in (1,6)-beta-D-glucan synthesis pathway"
PYK2	2.6	"Pyruvate kinase, glucose-repressed isoform"
GPH1	7.0	glycogen phosphorylase
**Lipid, fatty acid and isoprenoid metabolism**		
SWH1	2.6	Similar to mammalian oxysterol-binding protein
SHM1	2.0	"Serine hydroxymethyltransferase, mitochondrial"
GPD1	2.1	glycerol-3-phosphate dehydrogenase
GPI13	2.0	ER membrane localized phosphoryltransferase that adds phosphoethanolamine onto the third mannose residue of the glycosylphosphatidylinositol (GPI) anchor precursor; similar to human PIG-O protein
HMG2	2.1	3-hydroxy-3-methylglutaryl-coenzyme A (HMG-CoA) reductase isozyme
YML059C	2.1	questionable protein
DAK1	2.0	dihydroxyacetone kinase (putative)
INP53	2.0	inositol polyphosphate 5-phosphatase
NCR1	2.1	transmembrane protein (putative)
FAS2	2.0	fatty acid synthase alpha subunit
PLC1	2.0	phosphoinositide-specific phospholipase C
**Metabolism of vitamins, cofactors, and prosthetic groups**		
PYC2	2.5	pyruvate carboxylase
SHM1	2.0	"Serine hydroxymethyltransferase, mitochondrial"
BPL1	2.2	biotin:apoprotein ligase
PYC1	2.5	pyruvate carboxylase
ECM38	2.2	gamma-glutamyltransferase homolog
GCV2	2.3	glycine cleavage system P subunit|glycine decarboxylase complex P subunit|glycine synthase P subunit
FAS2	2.0	fatty acid synthase alpha subunit
**Secondary metabolism**		
ENO1	2.1	enolase I

Gene Name	Fold change	Gene product
GRE3	2.0	aldose reductase
GPI13	2.0	ER membrane localized phosphoryltransferase that adds phosphoethanolamine onto the third mannose residue of the glycosylphosphatidylinositol (GPI) anchor precursor; similar to human PIG-O protein
ARG1	2.0	arginosuccinate synthetase
**Energy**		
CDC19	2.6	pyruvate kinase
TPS1	2.6	trehalose-6-phosphate synthase/phosphatase complex 56 kDa synthase subunit
PYC2	2.5	pyruvate carboxylase
GLK1	3.0	glucokinase
NTH1	2.3	neutral trehalase
TPS2	2.2	trehalose-6-phosphate phosphatase
PDA1	2.2	pyruvate dehydrogenase alpha subunit (E1 alpha)
HXK1	5.2	hexokinase I (PI) (also called hexokinase A)
PYC1	2.5	pyruvate carboxylase
SOL4	2.8	6-phosphogluconolactonase
ENO1	2.1	enolase I
KGD1	2.4	alpha-ketoglutarate dehydrogenase
RAV1	2.1	Regulator of (H+)-ATPase in vacuolar membrane
ATP2	2.4	F(1)F(0)-ATPase complex beta subunit
SDH1	2.2	succinate dehydrogenase flavoprotein subunit
CSF1	2.2	Protein required for fermentation at low temperature
PDC5	2.3	pyruvate decarboxylase
GSY2	2.2	glycogen synthase (UDP-glucose-starch glucosyltransferase)
FKS1	2.6	"1,3-beta-D-glucan synthase"
TSL1	4.7	similar to TPS3 gene product|trehalose-6-phosphate synthase/phosphatase complex 123 kDa regulatory subunit
PGM2	4.1	phosphoglucomutase
MYO5	2.0	myosin I
TPS3	3.1	trehalose-6-phosphate synthase/phosphatase complex 115 kDa regulatory subunit
LAT1	2.5	pyruvate dehydrogenase complex dihydrolipoamide acetyltransferase component (E2)
ZWF1	3.3	glucose-6-phosphate dehydrogenase
CYT1	2.0	cytochrome c1
LSC1	2.0	"alpha subunit of succinyl-CoA ligase (synthetase; ATP-forming), a mitochondrial enzyme of the TCA cycle"
KRE5	2.2	"appears to function early in (1,6)-beta-D-glucan synthesis pathway"
PYK2	2.6	"Pyruvate kinase, glucose-repressed isoform"
GDH1	2.0	NADP-specific glutamate dehydrogenase
GPH1	7.0	glycogen phosphorylase
**Cell cycle and DNA processing**		
SWH1	2.6	Similar to mammalian oxysterol-binding protein
RPG1	2.2	translation initiation factor eIF3 subunit
MEC1	2.4	"Required for mitotic growth, DNA repair and mitotic recombination, regulates phosporylation of Rad53p, required for dmc1 arrest and meiotic recombination, homolog of fission yeast Rad3 and human ATR"
SDS24	2.1	"Similar to S. pombe SDS23, suppresses DIS2, localized to the nucleus"
BUD3	2.2	"Protein involved in bud-site selection and required for axial budding pattern; localizes with septins to bud neck in mitosis and may constitute """"axial landmark"""" for next round of budding"
SSD1	2.0	Product of gene unknown
TOM1	2.0	"hect-domain-containing protein, containing kinase motifs|similar to Rsp5"

Gene Name	Fold change	Gene product
SEC3	2.0	"Profilin synthetic lethal protein, has region of coiled-coil structure; subunit of the Exocyst complex--the Exocyst complex contains the gene products encoded by SEC3, SEC5, SEC6, SEC8, SEC10, SEC15 and EXO70 and is required for exocytosis"
PTC2	2.1	protein phosphatase type 2C
RSP5	2.5	involved in ubiquitin-mediated protein degradation
CHD1	2.3	transcriptional regulator
KEM1	2.2	5'-3' exonuclease
SAE2	2.2	Involved in meiotic recombination and chromosome metabolism
SCH9	2.1	cAMP-dependent protein kinase homolog|suppressor of cdc25ts
SIM1	2.1	(putative) invovled in control of DNA replication
PAN1	2.6	Involved in actin organization and endocytosis
CYR1	2.5	adenylate cyclase
NET1	2.0	Nucleolar protein involved in exit from mitosis
SCP160	2.2	"May be required during cell division for faithful partitioning of the ER-nuclear envelope membranes, involved in control of mitotic chromsome transmission"
JSN1	2.1	benomyl dependent tubulin mutant
ECM38	2.2	gamma-glutamyltransferase homolog
MSC1	2.2	"Protein of unknown function, green fluorescent protein (GFP)-fusion protein localizes to the endoplasmic reticulum; msc1 mutants are defective in directing meiotic recombination events to homologous chromatids"
FAR11	2.5	"Protein involved in G1 cell cycle arrest in response to pheromone, in a pathway different from the Far1p-dependent pathway; interacts with Far3p, Far7p, Far8p, Far9p, and Far10p"
APC1	2.1	ubiquitin ligase subunit
ATG2	2.1	peripheral membrane protein
BNI1	2.0	"formin, involved in spindle orientation"
NFI1	2.0	chromatin protein
WTM1	2.0	transcriptional modulator
PLC1	2.0	phosphoinositide-specific phospholipase C

**Transcription**		
SRB8	2.7	activation mediator subcomplex of RNA polymerase I holoenzyme
TUP1	2.0	"glucose repression regulatory protein, exhibits similarity to beta subunits of G proteins"
STP4	2.3	Involved in pre-tRNA splicing and in uptake of branched-chain amino acids
ADR1	2.7	positive transcriptional regulator
NPL3	3.6	contains RNA recognition motif|nuclear shuttling protein
SSN2	2.2	transcription factor
GAL83	2.0	Glucose repression protein
CHD1	2.3	transcriptional regulator
PAB1	2.5	poly(A) binding protein
ARC1	2.0	"Protein that binds tRNA and methionyl- and glutamyl-tRNA synthetases (Mes1p and Ygl245wp), delivering tRNA to them, stimulating catalysis, and ensuring their localization to the cytoplasm; also binds quadruplex nucleic acids"
KEM1	2.2	5'-3' exonuclease
SOL4	2.8	6-phosphogluconolactonase
MGA1	2.1	similar to heat shock transcription factor
BCY1	2.0	cAMP-dependent protein kinase regulatory subunit
TAO3	2.2	Identified in a hunt for mutants that activate OCH1 transcription
NET1	2.0	Nucleolar protein involved in exit from mitosis
TPK1	2.0	cAMP-dependent protein kinase catalytic subunit (putative)
JSN1	2.1	benomyl dependent tubulin mutant
GSP1	2.1	GTP-binding protein
WTM1	2.0	transcriptional modulator

Gene Name	Fold change	Gene product
**Protein synthesis**		
RPG1	2.2	translation initiation factor eIF3 subunit
EFT2	1.9	translation elongation factor 2 (EF-2)
PAB1	2.5	poly(A) binding protein
FRS2	2.0	phenylalanine-tRNA ligase subunit
ARC1	2.0	"Protein that binds tRNA and methionyl- and glutamyl-tRNA synthetases (Mes1p and Ygl245wp), delivering tRNA to them, stimulating catalysis, and ensuring their localization to the cytoplasm; also binds quadruplex nucleic acids"
YGR054W	2.5	yeast homolog of mammalian eIF2A
MES1	2.3	methionine-tRNA ligase
DED81	2.2	asparaginyl-tRNA synthetase
APE2	3.4	aminopeptidase yscII
EAP1	2.3	functionally analogous to mammalian 4E-BPsfunctional and limited sequence similarity to CAF20
DPS1	2.7	aspartyl-tRNA synthetase
EFT1	2.1	translation elongation factor 2 (EF-2)
CAM1	2.5	calcium and phospholipid binding protein homologous to translation elongation factor 1-gamma (EF-1gamma)
**Protein fate**		
SLA1	2.3	cytoskeletal protein binding protein
MEC1	2.4	"Required for mitotic growth, DNA repair and mitotic recombination, regulates phosporylation of Rad53p, required for dmc1 arrest and meiotic recombination, homolog of fission yeast Rad3 and human ATR"
SSE2	2.8	HSP70 family|SSE1 homolog
SDS24	2.1	"Similar to S. pombe SDS23, suppresses DIS2, localized to the nucleus"
APE3	2.1	aminopeptidase yscIII
YCL044C	2.4	questionable protein
ABP1	2.6	actin binding protein
BPL1	2.2	biotin:apoprotein ligase
KIN1	2.0	Serine/threonine protein kinase
NPL3	3.6	contains RNA recognition motif|nuclear shuttling protein
TOM1	2.0	"hect-domain-containing protein, containing kinase motifs|similar to Rsp5"
GDA1	2.5	guanosine diphosphatase of Golgi membrane
PRB1	2.3	vacuolar protease B
GAL83	2.0	Glucose repression protein
PTC2	2.1	protein phosphatase type 2C
KAP123	2.2	karyopherin beta 4
RSP5	2.5	involved in ubiquitin-mediated protein degradation
BLM3	2.0	involved in protecting the cell against bleomycin damage
CHC1	2.3	Clathrin heavy chain
UBR1	2.3	ubiquitin-protein ligase
DIE2	2.3	glucosyltransferase
ECM29	2.0	ExtraCellular Mutant
KSP1	2.6	Serine/threonine kinase similar to casein kinase II and other serine/threonine protein kinases
SCH9	2.1	cAMP-dependent protein kinase homolog|suppressor of cdc25ts
SEC24	2.5	vesicle coat component
PAN1	2.6	Involved in actin organization and endocytosis
TPK1	2.0	cAMP-dependent protein kinase catalytic subunit (putative)
SSC1	2.4	mitochondrial matrix protein involved in protein import|Endo.SceI endonuclease subunit
PMT4	2.0	dolichyl phosphate-D-mannose:protein O-D-mannosyltransferase
APE2	3.4	aminopeptidase yscII
HSP104	2.4	heat shock protein 104

Gene Name	Fold change	Gene product
GPI13	2.0	ER membrane localized phosphoryltransferase that adds phosphoethanolamine onto the third mannose residue of the glycosylphosphatidylinositol (GPI) anchor precursor; similar to human PIG-O protein
VPS13	2.6	homologous to human COH1
UBR2	2.1	ubiquitin-protein ligase (E3)
YPS1	2.0	GPI-anchored aspartic protease
ECM38	2.2	gamma-glutamyltransferase homolog
SEC61	2.5	membrane component of ER protein translocation apparatus
STE23	2.0	involved in a-factor processing
YML059C	2.1	questionable protein
TOM40	2.4	forms the outer membrane import channel|mitochondrial outer membrane protein
UBP15	2.5	deubiquitinating enzyme (putative)
APC1	2.1	ubiquitin ligase subunit
ATG2	2.1	peripheral membrane protein
YNR047W	2.1	questionable protein
STI1	2.2	heat shock protein also induced by canavanine and entry into stationary phase
VPS17	2.3	Peripheral membrane protein required for vacuolar protein sorting
NFI1	2.0	chromatin protein
KRE5	2.2	"appears to function early in (1,6)-beta-D-glucan synthesis pathway"
BRO1	2.2	BCK1-like resistance to osmotic shock
SEC16	2.7	vesicle coat component
SSE1	2.2	HSP70 family|SSA1 SSE2 homolog
PEP4	2.2	vacuolar proteinase A
FAS2	2.0	fatty acid synthase alpha subunit
APM1	2.2	clathrin associated protein complex medium subunit
**Regulation of metabolism and protein function**		
IRA1	2.0	GTPase activating protein (GAP)
ILV6	2.6	"Regulatory subunit of acetolactate synthase, which catalyzes the first step of branched-chain amino acid biosynthesis; enhances activity of the Ilv2p catalytic subunit, localizes to mitochondria"
SEC7	2.0	guanine nucleotide exchange protein for ARF
PTC2	2.1	protein phosphatase type 2C
BLM3	2.0	involved in protecting the cell against bleomycin damage
PIL1	2.4	Phosphorylation Inhibited by Long chain base
BCY1	2.0	cAMP-dependent protein kinase regulatory subunit
SSC1	2.4	mitochondrial matrix protein involved in protein import|Endo.SceI endonuclease subunit
TSL1	4.7	similar to TPS3 gene product|trehalose-6-phosphate synthase/phosphatase complex 123 kDa regulatory subunit
IRA2	2.3	GTPase activating protein|highly homologous to Ira1p|neurofibromin homolog
STI1	2.2	heat shock protein also induced by canavanine and entry into stationary phase
WTM1	2.0	transcriptional modulator
**Cellular transport, transport facilities and transport routes**		
SLA1	2.3	cytoskeletal protein binding protein
FUI1	2.0	uridine permease
EDE1	2.9	EH domain protein involved in endocytosis
SEC18	2.1	ATPase|NSF|protein involved in protein transport between ER and Golgi
VMA2	2.1	vacuolar ATPase V1 domain subunit B (60 kDa)
SDS24	2.1	"Similar to S. pombe SDS23, suppresses DIS2, localized to the nucleus"
GLK1	3.0	glucokinase
STP4	2.3	Involved in pre-tRNA splicing and in uptake of branched-chain amino acids

Gene Name	Fold change	Gene product
COP1	2.0	coatomer complex gamma-alpha-COP alpha subunit
SEC7	2.0	guanine nucleotide exchange protein for ARF
RVS167	2.0	cytoskeletal protein (putative)
PDR15	2.0	multidrug resistance transporter (putative)
NPL3	3.6	contains RNA recognition motif|nuclear shuttling protein
NHX1	2.1	Na+/H+ exchanger
TOM1	2.0	"hect-domain-containing protein, containing kinase motifs|similar to Rsp5"
SEC3	2.0	"Profilin synthetic lethal protein, has region of coiled-coil structure; subunit of the Exocyst complex--the Exocyst complex contains the gene products encoded by SEC3, SEC5, SEC6, SEC8, SEC10, SEC15 and EXO70 and is required for exocytosis"
KAP123	2.2	karyopherin beta 4
PAB1	2.5	poly(A) binding protein
DNF1	2.1	Potential aminophospholipid translocase
RET2	1.9	coatomer (COPI) complex delta subunit
ARC1	2.0	"Protein that binds tRNA and methionyl- and glutamyl-tRNA synthetases (Mes1p and Ygl245wp), delivering tRNA to them, stimulating catalysis, and ensuring their localization to the cytoplasm; also binds quadruplex nucleic acids"
CHC1	2.3	Clathrin heavy chain
NEO1	2.1	P-type ATPase
SEC24	2.5	vesicle coat component
PAN1	2.6	Involved in actin organization and endocytosis
SCP160	2.2	"May be required during cell division for faithful partitioning of the ER-nuclear envelope membranes, involved in control of mitotic chromsome transmission"
SSC1	2.4	mitochondrial matrix protein involved in protein import|Endo.SceI endonuclease subunit
MIR1	2.2	Product of gene unknown
ECM27	2.3	ExtraCellular Mutant
ATP2	2.4	F(1)F(0)-ATPase complex beta subunit
SDH1	2.2	succinate dehydrogenase flavoprotein subunit
GAP1	2.6	general amino acid permease
VPS13	2.6	homologous to human COH1
YBT1	2.1	"Yeast bile transporter, similar to mammalian bile transporter"
GSP1	2.1	GTP-binding protein
VRP1	2.7	proline-rich protein verprolin
SEC61	2.5	membrane component of ER protein translocation apparatus
HXT2	2.1	high affinity hexose transporter-2
MYO5	2.0	myosin I
SSO2	2.1	t-SNARE
TOM40	2.4	forms the outer membrane import channel|mitochondrial outer membrane protein
ATG2	2.1	peripheral membrane protein
BNI1	2.0	"formin, involved in spindle orientation"
SEC21	2.0	PEST sequence-containing protein|non-clathrin coat protein
PHO91	2.0	Low-affinity phosphate transporter
CYT1	2.0	cytochrome c1
INP53	2.0	inositol polyphosphate 5-phosphatase
VPS17	2.3	Peripheral membrane protein required for vacuolar protein sorting
GSP2	2.1	GTP-binding protein|Gsp1p homolog
NCR1	2.1	transmembrane protein (putative)
SVL3	2.1	"Protein of unknown function, mutant phenotype suggests a potential role in vacuolar function; green fluorescent protein (GFP)-fusion protein localizes to the cell periphery, cytoplasm, bud, and bud neck"
BRO1	2.2	BCK1-like resistance to osmotic shock
SEC16	2.7	vesicle coat component
APM1	2.2	clathrin associated protein complex medium subunit

Gene Name	Fold change	Gene product
**Cellular communication/Signal transduction mechanism**		
IRA1	2.0	GTPase activating protein (GAP)
GAL83	2.0	Glucose repression protein
PTC2	2.1	protein phosphatase type 2C
BCY1	2.0	cAMP-dependent protein kinase regulatory subunit
CYR1	2.5	adenylate cyclase
TPK1	2.0	cAMP-dependent protein kinase catalytic subunit (putative)
GIS3	2.0	GIG3 suppressor
SRV2	2.4	70 kDa adenylyl cyclase-associated protein
BNI1	2.0	"formin, involved in spindle orientation"
IRA2	2.3	GTPase activating protein|highly homologous to Ira1p|neurofibromin homolog
INP53	2.0	inositol polyphosphate 5-phosphatase
BRO1	2.2	BCK1-like resistance to osmotic shock
PLC1	2.0	phosphoinositide-specific phospholipase C
**Cell rescue, defense and virulence**		
IST2	2.5	"Plasma membrane protein that may be involved in osmotolerance, localizes to the mother cell in small-budded cells and to the bud in medium- and large-budded cells; mRNA is transported to the bud tip by an actomysin-driven process"
TPS1	2.6	trehalose-6-phosphate synthase/phosphatase complex 56 kDa synthase subunit
MEC1	2.4	"Required for mitotic growth, DNA repair and mitotic recombination, regulates phosporylation of Rad53p, required for dmc1 arrest and meiotic recombination, homolog of fission yeast Rad3 and human ATR"
SSE2	2.8	HSP70 family|SSE1 homolog
GPD1	2.1	glycerol-3-phosphate dehydrogenase
NTH1	2.3	neutral trehalase
TPS2	2.2	trehalose-6-phosphate phosphatase
SED1	3.5	cell surface glycoprotein (putative)
SSD1	2.0	Product of gene unknown
RVS167	2.0	cytoskeletal protein (putative)
NHX1	2.1	Na+/H+ exchanger
PRB1	2.3	vacuolar protease B
PTC2	2.1	protein phosphatase type 2C
RSP5	2.5	involved in ubiquitin-mediated protein degradation
BLM3	2.0	involved in protecting the cell against bleomycin damage
PIL1	2.4	Phosphorylation Inhibited by Long chain base
MGA1	2.1	similar to heat shock transcription factor
GRE3	2.0	aldose reductase
SCH9	2.1	cAMP-dependent protein kinase homolog|suppressor of cdc25ts
BCY1	2.0	cAMP-dependent protein kinase regulatory subunit
SSC1	2.4	mitochondrial matrix protein involved in protein import|Endo.SceI endonuclease subunit
HSP104	2.4	heat shock protein 104
VRP1	2.7	proline-rich protein verprolin
DAK1	2.0	dihydroxyacetone kinase (putative)
TSL1	4.7	similar to TPS3 gene product|trehalose-6-phosphate synthase/phosphatase complex 123 kDa regulatory subunit
MYO5	2.0	myosin I
GAD1	2.2	glutamate decarboxylase
TPS3	3.1	trehalose-6-phosphate synthase/phosphatase complex 115 kDa regulatory subunit
YNL190W	2.0	questionable protein
ZWF1	3.3	glucose-6-phosphate dehydrogenase
BNI1	2.0	"formin, involved in spindle orientation"

Gene Name	Fold change	Gene product
STI1	2.2	heat shock protein also induced by canavanine and entry into stationary phase
BRO1	2.2	BCK1-like resistance to osmotic shock
SSE1	2.2	HSP70 family|SSA1 SSE2 homolog
PEP4	2.2	vacuolar proteinase A
**Interaction with the environment**		
VMA2	2.1	vacuolar ATPase V1 domain subunit B (60 kDa)
IRA1	2.0	GTPase activating protein (GAP)
GPD1	2.1	glycerol-3-phosphate dehydrogenase
NHX1	2.1	Na+/H+ exchanger
GAL83	2.0	Glucose repression protein
PTC2	2.1	protein phosphatase type 2C
PIL1	2.4	Phosphorylation Inhibited by Long chain base
SCH9	2.1	cAMP-dependent protein kinase homolog|suppressor of cdc25ts
BCY1	2.0	cAMP-dependent protein kinase regulatory subunit
CYR1	2.5	adenylate cyclase
PRY3	2.6	"Pathogen Related in Sc, contains homology to the plant PR-1 class of pathogen related proteins. The protein sequence is over 60% identical with the Pry2p & Pry3p over 145 resid. PRY1 is >35% identical (50% similar) to tobacco PR-1c protein."
SCP160	2.2	"May be required during cell division for faithful partitioning of the ER-nuclear envelope membranes, involved in control of mitotic chromosome transmission"
RAV1	2.1	Regulator of (H+)-ATPase in vacuolar membrane
MIR1	2.2	Product of gene unknown
ATP2	2.4	F(1)F(0)-ATPase complex beta subunit
GFA1	3.2	glucoseamine-6-phosphate synthase|glutamine_fructose-6-phosphate amidotransferase
STE23	2.0	involved in a-factor processing
MYO5	2.0	myosin I
FAR11	2.5	"Protein involved in G1 cell cycle arrest in response to pheromone, in a pathway different from the Far1p-dependent pathway; interacts with Far3p, Far7p, Far8p, Far9p, and Far10p"
BNI1	2.0	"formin, involved in spindle orientation"
PHO91	2.0	Low-affinity phosphate transporter
YNR047W	2.1	questionable protein
BRO1	2.2	BCK1-like resistance to osmotic shock
SSE1	2.2	HSP70 family|SSA1 SSE2 homolog
**Transposable elements, viral and plasmid proteins**		
YDR170W-A	2.4	Ty ORF
YDR210W-A	2.9	TyA Gag protein; the main structural constituent of virus-like particles (VLPs)
YDR261W-A	2.4	TyA Gag protein; the main structural constituent of virus-like particles (VLPs)
YGR161W-A	2.1	TyA Gag protein; the main structural constituent of virus-like particles (VLPs)
YLR410W-A	2.8	TyA Gag protein; the main structural constituent of virus-like particles (VLPs)
YOR192C-A	2.8	TyA Gag protein; the main structural constituent of virus-like particles (VLPs)
YOR343W-A	3.0	TyA Gag protein; the main structural constituent of virus-like particles (VLPs)
YOR343W-B	3.3	"TyB Gag-Pol protein; proteolytically processed to make the Gag, RT, PR, and IN proteins that are required for retrotransposition"
**Cell fate**		
SLA1	2.3	cytoskeletal protein binding protein
ABP1	2.6	actin binding protein
WHI4	2.2	RNA binding protein (putative)|WHI3 homolog

Gene Name	Fold change	Gene product
SSD1	2.0	Product of gene unknown
RVS167	2.0	cytoskeletal protein (putative)
SCH9	2.1	cAMP-dependent protein kinase homolog|suppressor of cdc25ts
BCY1	2.0	cAMP-dependent protein kinase regulatory subunit
SIM1	2.1	(putative) invovled in control of DNA replication
TAO3	2.2	Identified in a hunt for mutants that activate OCH1 transcription
ATP2	2.4	F(1)F(0)-ATPase complex beta subunit
VRP1	2.7	proline-rich protein verprolin
SSO2	2.1	t-SNARE
SRV2	2.4	70 kDa adenylyl cyclase-associated protein
WHI3	2.0	RNA binding protein (putative)
BNI1	2.0	"formin, involved in spindle orientation"
STI1	2.2	heat shock protein also induced by canavanine and entry into stationary phase
NFI1	2.0	chromatin protein
KRE5	2.2	"appears to function early in (1,6)-beta-D-glucan synthesis pathway"
SVL3	2.1	"Protein of unknown function, mutant phenotype suggests a potential role in vacuolar function; green fluorescent protein (GFP)-fusion protein localizes to the cell periphery, cytoplasm, bud, and bud neck"
SSE1	2.2	HSP70 family|SSA1 SSE2 homolog
PLC1	2.0	phosphoinositide-specific phospholipase C
**Biogenesis of cellular components**		
SLA1	2.3	cytoskeletal protein binding protein
YCL044C	2.4	questionable protein
ABP1	2.6	actin binding protein
SED1	3.5	cell surface glycoprotein (putative)
ADR1	2.7	positive transcriptional regulator
SSD1	2.0	Product of gene unknown
RVS167	2.0	cytoskeletal protein (putative)
TOM1	2.0	"hect-domain-containing protein, containing kinase motifs|similar to Rsp5"
CWH41	2.1	glucosidase I
ECM29	2.0	ExtraCellular Mutant
BCY1	2.0	cAMP-dependent protein kinase regulatory subunit
SIM1	2.1	(putative) invovled in control of DNA replication
PAN1	2.6	Involved in actin organization and endocytosis
BBC1	2.8	shows synthetic fitness defect with bni1 mutants and associates with the Bee1p-Vrp1p-Myo3/5p complex
MHP1	2.5	microtubule-associated protein (MAP) (putative)
NET1	2.0	Nucleolar protein involved in exit from mitosis
ECM27	2.3	ExtraCellular Mutant
CWP1	3.4	cell wall mannoprotein
GFA1	3.2	glucoseamine-6-phosphate synthase|glutamine_fructose-6-phosphate amidotransferase
SDH1	2.2	succinate dehydrogenase flavoprotein subunit
ECM4	2.4	ExtraCellular Mutant
MMM1	2.4	mitochondrial outer membrane protein
GSP1	2.1	GTP-binding protein
ECM38	2.2	gamma-glutamyltransferase homolog
VRP1	2.7	proline-rich protein verprolin
FKS1	2.6	"1,3-beta-D-glucan synthase"
MYO5	2.0	myosin I
SSO2	2.1	t-SNARE
SRV2	2.4	70 kDa adenylyl cyclase-associated protein
ATG2	2.1	peripheral membrane protein
BNI1	2.0	"formin, involved in spindle orientation"
YOL155C	2.1	ORF

Gene Name	Fold change	Gene product
DFG16	2.1	"Probable multiple transmembrane protein, involved in invasive growth upon nitrogen starvation"
INP53	2.0	inositol polyphosphate 5-phosphatase
NFI1	2.0	chromatin protein
GSP2	2.1	GTP-binding protein|Gsp1p homolog
SVL3	2.1	"Protein of unknown function, mutant phenotype suggests a potential role in vacuolar function; green fluorescent protein (GFP)-fusion protein localizes to the cell periphery, cytoplasm, bud, and bud neck"
APM1	2.2	clathrin associated protein complex medium subunit
PLC1	2.0	phosphoinositide-specific phospholipase C
**Cell type differentiation**		
SLA1	2.3	cytoskeletal protein binding protein
IRA1	2.0	GTPase activating protein (GAP)
BUD3	2.2	"Protein involved in bud-site selection and required for axial budding pattern; localizes with septins to bud neck in mitosis and may constitute """"axial landmark"""" for next round of budding"
ABP1	2.6	actin binding protein
SPO71	2.0	"Meiosis-specific protein of unknown function, required for spore wall formation during sporulation; dispensible for both nuclear divisions during meiosis"
SSD1	2.0	Product of gene unknown
RVS167	2.0	cytoskeletal protein (putative)
EMI2	2.7	Early Meiotic Induction
PRB1	2.3	vacuolar protease B
SEC3	2.0	"Profilin synthetic lethal protein, has region of coiled-coil structure; subunit of the Exocyst complex--the Exocyst complex contains the gene products encoded by SEC3, SEC5, SEC6, SEC8, SEC10, SEC15 and EXO70 and is required for exocytosis"
GAL83	2.0	Glucose repression protein
RSP5	2.5	involved in ubiquitin-mediated protein degradation
KEM1	2.2	5'-3' exonuclease
MGA1	2.1	similar to heat shock transcription factor
BCY1	2.0	cAMP-dependent protein kinase regulatory subunit
TAO3	2.2	Identified in a hunt for mutants that activate OCH1 transcription
PAN1	2.6	Involved in actin organization and endocytosis
CYR1	2.5	adenylate cyclase
TPK1	2.0	cAMP-dependent protein kinase catalytic subunit (putative)
VRP1	2.7	proline-rich protein verprolin
MYO5	2.0	myosin I
SSO2	2.1	t-SNARE
SRV2	2.4	70 kDa adenylyl cyclase-associated protein
WHI3	2.0	RNA binding protein (putative)
ATG2	2.1	peripheral membrane protein
BNI1	2.0	"formin, involved in spindle orientation"
DFG16	2.1	"Probable multiple transmembrane protein, involved in invasive growth upon nitrogen starvation"
NFI1	2.0	chromatin protein
SVL3	2.1	"Protein of unknown function, mutant phenotype suggests a potential role in vacuolar function; green fluorescent protein (GFP)-fusion protein localizes to the cell periphery, cytoplasm, bud, and bud neck"
PEP4	2.2	vacuolar proteinase A
PLC1	2.0	phosphoinositide-specific phospholipase C
**Unclassified proteins**		
YAL053W	2.2	Protein of unknown function; green fluorescent protein (GFP)-fusion protein localizes to the cytoplasm in a punctate pattern
YBR108W	2.3	questionable protein
YCL045C	2.1	questionable protein

Gene Name	Fold change	Gene product
SYP1	2.1	Suppressor of Yeast Profilin deletion
YCR061W	2.7	Protein of unknown function; green fluorescent protein (GFP)-fusion protein localizes to the cytoplasm in a punctate pattern
YEL043W	2.3	questionable protein
ZRG8	2.5	Zinc regulated gene
SCY1	2.0	Suppressor of GTPase mutant
YHR087W	3.8	questionable protein
YHR214W-A	2.0	questionable protein
YIL169C	2.4	questionable protein
YKR018C	2.3	questionable protein
YLR162W	2.1	questionable protein
YLR187W	2.1	"Protein of unknown function; green fluorescent protein (GFP)-fusion protein localizes to the cell periphery, cytoplasm, bud, and bud neck"
YML072C	2.1	Protein of unknown function; green fluorescent protein (GFP)-fusion protein localizes to the cell periphery
YMR086W	2.2	Protein of unknown function; green fluorescent protein (GFP)-fusion protein localizes to the cell periphery
YMR196W	2.2	questionable protein
YMR247C	2.1	questionable protein
YNL321W	2.1	not essential for vegetative growth
CUE5	2.0	"Protein containing a CUE domain that binds ubiquitin, which may facilitate intramolecular monoubiquitination; green fluorescent protein (GFP)-fusion protein localizes to the cytoplasm in a punctate pattern"
YOR066W	2.0	questionable protein
YPL137C	2.1	questionable protein
YPL184C	2.1	questionable protein
