# Supplementary material for: Quercetin Protects Saccharomyces cerevisiae against Oxidative Stress by Inducing Trehalose Biosynthesis and the Cell Wall Integrity Pathway
Source: PLoS One. 2012 Sep 18;7(9):e45494. doi: 10.1371/journal.pone.0045494 (PMC3445532; doi:10.1371/journal.pone.0045494)
Supplement: Table S2 — Genes down regulated in quercetin treated cells. (PDF) [file pone.0045494.s002.pdf]

Table S2 Genes down regulated in quercetin treated cells. *S. cerevisiae* BY4741 was grown to exponential phase in YPD medium and treated with 300 µM of quercetin or equal volume of DMSO (control cells) for 15 min. Genes differentially regulated (quercetin *vs* control) were sorted into functional categories according to MIPS; some genes were listed in more than one category.

Gene name	Fold change	Gene Product
**Amino acid metabolism**		
SRB6	-2.1	part of Srb/mediator complex; transcription factor
CIT2	-1.0	citrate synthase
MAK31	-1.8	member of the Sm protein family
DTD1	-1.2	D-Tyr-tRNA(Tyr) deacylase
LYS5	-1.0	alpha aminoadipate reductase phosphopantetheinyl transferase
YIL167W	-1.3	questionable protein
MET14	-1.1	adenylylsulfate kinase
MET1	-1.1	S-adenosyl-L-methionine uroporphyrinogen III transmethylase
ASP3-1	-1.2	nitrogen catabolite-regulated cell-wall L-asparaginase II
ASP3-2	-1.0	nitrogen catabolite-regulated cell-wall L-asparaginase II
ASP3-3	-1.0	nitrogen catabolite-regulated cell-wall L-asparaginase II
ASP3-4	-1.2	nitrogen catabolite-regulated cell-wall L-asparaginase II
ORT1	-1.0	ornithine transporter of the mitochondrial inner membrane
MET31	-1.2	highly homologous to Met32p; transcriptional regulator of sulfur amino acid metabolism
**Nucleotide/nucleoside/nucleobase metabolism**		
RIB1	-0.9	GTP cyclohydrolase II
DUT1	-1.2	dUTP pyrophosphatase
CDC36	-1.1	basal transcription inhibitor; transcriptional regulator
HPT1	-1.8	hypoxanthine guanine phosphoribosyltransferase
GNA1	-1.2	glucosamine-phosphate N-acetyltransferase
SLX9	-0.9	protein of unknown function
RRP46	-1.2	3'->5' exoribonuclease
CPD1	-1.9	2',3'-cyclic nucleotide 3'-phosphodiesterase
DAL7	-1.5	malate synthase 2
MRT4	-1.3	mRna turnover 4
PUS5	-1.8	RNA:pseudouridine (Psi)-synthase
URA10	-0.9	orotate phosphoribosyltransferase 2
LSM7	-1.6	snRNP protein
DCP1	-0.9	mRNA decapping enzyme
SPS4	-1.6	sporulation-specific protein
FCY1	-1.2	cytosine deaminase
**Phosphate metabolism**		
DUT1	-1.2	dUTP pyrophosphatase
YPI1	-1.0	PP1 phosphatase inhibitor
DOG2	-0.9	2-deoxyglucose-6-phosphate phosphatase
MET14	-1.1	adenylylsulfate kinase
FBP1	-0.9	fructose-1,6-bisphosphatase
CTL1	-1.3	RNA triphosphatase
**Regulation of phosphate metabolism**		
YPI1	-1.0	PP1 phosphatase inhibitor
**C-compound and carbohydrate metabolism**		
ACS1	-1.5	acetyl CoA synthetase
LDB7	-0.9	protein involved in mannosylphosphorylation of cell wall mannoproteins

Gene name	Fold change	Gene Product
HAP3	-1.3	transcriptional activator protein of CYC1 (component of HAP2/HAP3 heteromer)
GAL10	-1.2	UDP-glucose 4-epimerase
SRB6	-2.1	part of Srb/Mediator complex; transcription factor
CIT2	-1.0	citrate synthase
OST4	-0.9	oligosaccharyltransferase subunit
SOR2	-1.3	protein of unknown function, possible role in fructose or mannose metabolism
NRG1	-1.6	binds to UAS-1 in the STA1 promoter and can interact with Ssn6; transcriptional repressor
SNF11	-1.4	SWI/SNF global transcription activator complex component
YDR248C	-1.2	questionable protein
GPI11	-1.3	protein involved in glycosylphosphatidylinositol (GPI) biosynthesis
ACN9	-1.2	Protein of the mitochondrial intermembrane space, required for acetate utilization and gluconeogenesis
FAU1	-1.1	5,10-methenyltetrahydrofolate synthetase
GNA1	-1.2	glucosamine-phosphate N-acetyltransferase
SNF6	-0.9	chromatin remodeling Snf/Swi complex subunit
DOG2	-0.9	2-deoxyglucose-6-phosphate phosphatase
DAL7	-1.5	malate synthase 2
GON7	-1.4	protein proposed to be involved in the transfer of mannosylphosphate groups onto N-linked oligosaccharides
SOR1	-1.5	sorbitol dehydrogenase, sorbitol-induced
KTI12	-1.1	elongator associated protein
YLR164W	-1.1	strong similarity to succinate dehydrogenase membrane anchor subunit Sdh4p
IDP3	-1.0	NADP-dependent isocitrate dehydrogenase
YNR071C	-1.3	questionable protein
RKI1	-0.9	ribose-5-phosphate ketol-isomerase
OST2	-1.4	oligosaccharyltransferase epsilon subunit
SPR1	-1.1	exo-1,3-beta-glucanase, sporulation-specific
YOR283W	-0.9	questionable protein
**Lipid, fatty acid and isoprenoid metabolism**		
RER2	-0.9	cis-prenyltransferase
GPI11	-1.3	protein involved in glycosylphosphatidylinositol (GPI) biosynthesis
YLL049W	-0.9	questionable protein
ARV1	-1.0	protein involved in sterol distribution
IDP3	-1.0	NADP-dependent isocitrate dehydrogenase
SPO1	-0.9	similar to phospholipase B
GPI15	-1.6	human Pig-H homolog
CYB5	-1.1	cytochrome b5
YOL101C	-0.9	questionable protein
INO4	-1.7	basic helix-loop-helix (bHLH) protein
YPL095C	-1.0	questionable protein
PPT2	-2.1	phosphopantetheine:protein transferase (PPTase)
DAP1	-1.5	sterol-binding protein (putative)
**Metabolism of vitamins, cofactors, and prosthetic groups**		
ACS1	-1.5	acetyl CoA synthetase
RIB1	-0.9	GTP cyclohydrolase II
PET18	-2.2	protein required for respiratory growth and stability of the mitochondrial genome
HEM3	-0.9	phorphobilinogen deaminase (uroporphyrinogen synthase)
FAU1	-1.1	5,10-methenyltetrahydrofolate synthetase
SNO3	-0.9	protein is related with B(1) biosynthesis during the exponential phase

Gene name	Fold change	Gene Product
LYS5	-1.0	alpha aminoadipate reductase phosphopantetheinyl transferase
QCR9	-1.0	ubiquinol cytochrome c oxidoreductase complex 7.3 kDa subunit 9
FMC1	-1.2	assembly factor of ATP synthase in heat stress
MET1	-1.1	S-adenosyl-L-methionine uroporphyrinogen III transmethylase
IDP3	-1.0	NADP-dependent isocitrate dehydrogenase
BIO3	-1.0	7,8-diamino-pelargonic acid aminotransferase (DAPA) aminotransferase
ISU1	-1.1	iron-sulfur cluster nifU-like protein
PPT2	-2.1	phosphopantetheine:protein transferase (PPTase)
**Secondary metabolism**		
KTI11	-1.2	similar to Hemiascomycetous yeast protein
MAK31	-1.8	member of the Sm protein family
HEM3	-0.9	phorphobilinogen deaminase (uroporphyrinogen synthase)
TSA2	-1.0	thioredoxin-peroxidase
YJR097W	-1.5	protein that may function as a cochaperone
MET1	-1.1	S-adenosyl-L-methionine uroporphyrinogen III transmethylase
**Energy**		
ACS1	-1.5	acetyl CoA synthetase
NRG2	-1.6	NRG1 homolog
CIT2	-1.0	citrate synthase
COX9	-2.0	cytochrome c oxidase subunit VIIa
INH1	-1.4	ATPase inhibitor
PET100	-1.5	cytochrome c oxidase-specific assembly factor
YDR115W	-1.4	questionable protein
TIM11	-1.7	mitochondrial F1F0-ATPase subunit e
ACN9	-1.2	Protein of the mitochondrial intermembrane space, required for acetate utilization and gluconeogenesis
QCR7	-1.1	ubiquinol cytochrome C oxidoreductase subunit 7
UBC8	-1.0	ubiquitin-conjugating enzyme; ubiquitin-protein ligase
YPI1	-1.0	PP1 phosphatase inhibitor
QCR6	-1.3	ubiquinol cytochrome C oxidoreductase subunit 6 (17 kDa)
YGL226W	-1.0	questionable protein
QCR9	-1.0	ubiquinol cytochrome c oxidoreductase complex 7.3 kDa subunit 9
VMA10	-1.2	vacuolar ATPase V1 domain subunit G (13 kDa)
VMA22	-1.9	required for V-ATPase activity
DAL7	-1.5	malate synthase 2
CYC1	-0.9	iso-1-cytochrome c
COX17	-1.5	cysteine-rich protein
COX12	-1.1	cytochrome c oxidase subunit VIb
YLR164W	-1.1	strong similarity to succinate dehydrogenase membrane anchor subunit Sdh4p
FBP1	-0.9	fructose-1,6-bisphosphatase
COX8	-1.0	cytochrome c oxidase chain VIII
ATP18	-1.7	ATP synthase associated protein
YML087C	-1.1	questionable protein
COX14	-1.2	mitochondrial membrane protein
MRPS17	-0.9	mitochondrial ribosomal protein of the small subunit
COX7	-2.2	cytochrome c oxidase subunit VII
YMR299C	-1.3	protein of unknown function; GFP-fusion protein localizes to the cytoplasm
IDP3	-1.0	NADP-dependent isocitrate dehydrogenase
ATP19	-1.2	ATP synthase subunit k homolog
RKI1	-0.9	ribose-5-phosphate ketol-isomerase
YOR283W	-0.9	questionable protein
OYE3	-1.8	NADPH dehydrogenase
ATP15	-1.3	ATP synthase epsilon subunit
ATP20	-1.5	ATP synthase subunit g homolog

Gene name	Fold change	Gene Product
MRPL51	-1.1	mitochondrial ribosomal protein of the large subunit
**Cell cycle and DNA processing**		
POP5	-1.3	RNase MRP subunit (putative); RNase P integral subunit
HTA2	-1.7	histone H2A
KTI11	-1.2	similar to Hemiascomycetous yeast protein
HHF1	-2.3	histone H4 (HHF1 and HHF2 code for identical proteins)
HHT1	-1.7	histone H3 (HHT1 and HHT2 code for identical proteins)
POL30	-1.1	proliferating Cell Nuclear Antigen (PCNA)
NHP6B	-1.2	11 kDa nonhistone chromosomal protein
SLX1	-1.3	subunit of a complex, with Slx4p, that hydrolyzes 5` branches from duplex DNA
DAD3	-1.3	outer kinetochore protein - part of Dam1 complex
DPB3	-1.1	DNA polymerase II C and C' subunits
HMLALPHA2	-2.7	mating type regulatory protein, silenced copy at HML
HTL1	-2.5	high-temperature lethal
MATALPHA2	-3.0	mating type regulatory protein, expressed copy at MAT locus
APC11	-1.2	anaphase promoting complex (APC) subunit
IWR1	-0.9	interacts with RNA Polymerase II
MSH5	-1.2	mutS homolog
CDC36	-1.1	basal transcription inhibitor; transcriptional regulator
RAD57	-1.2	RecA homolog; interacts with Rad 55p
DAD1	-1.7	Duo1 and Dam1 interacting; localized to intranuclear spindles and spindle pole bodies
SNF11	-1.4	SWI/SNF global transcription activator complex component
TFB5	-1.9	Component of general transcription and DNA repair factor TFIIH
STN1	-1.2	involved in telomere length regulation
TAF10	-1.2	TFIID subunit
SPC19	-1.2	spindle pole component
HTA1	-1.7	histone H2A (HTA1 and HTA2 code for nearly identical proteins)
MCM21	-0.9	Involved in minichromosome maintenance
DAD4	-1.7	outer kinetochore protein - part of Dam1 complex
SEM1	-2.6	Regulator of exocytosis and pseudohyphal differentiation
DYN2	-2.1	dynein light chain (putative)
ADA2	-1.4	general transcriptional adaptor or co-activator
BIM1	-2.0	binding to microtubules
YER066W	-1.2	questionable protein
ISC10	-1.7	meiosis-specific protein required for spore formation
GNA1	-1.2	glucosamine-phosphate N-acetyltransferase
FAR7	-1.0	protein involved in G1 cell cycle arrest in response to pheromone, in a pathway different from the Far1p-dependent pathway
SPT4	-3.1	transcription elongation protein
SLX9	-0.9	protein of unknown function
SYF2	-0.9	SYnthetic lethal with CDC40
TRX2	-1.0	thioredoxin
CPD1	-1.9	2',3'-cyclic nucleotide 3'-phosphodiesterase
SNF6	-0.9	chromatin remodeling Snf/Swi complex subunit
LRP1	-1.6	like rRNA Processing protein involved in regulation of DNA repair and recombination
YNG2	-1.2	NuA4 histone acetyltransferase complex component
SPO12	-1.5	sporulation protein
SPO16	-1.0	sporulation protein
SSP1	-1.2	essential for meiosis and spore formation
EST3	-1.8	Subunit of the telomerase
REV7	-1.5	DNA polymerase zeta (pol-zeta) subunit
STS1	-1.1	restores protein transport when overexpressed and rRNA stability to a sec23 mutation

Gene name	Fold change	Gene Product
YIR024C	-1.1	(putative) involved in cell cycle control
YJL065C	-1.0	subunit of ISW2/yCHRAC chromatin remodelling complex
NNF1	-1.3	spindle pole protein, required for accurate chromosome segregation
MCM22	-1.1	required for maintenance of chromosomes and minichromosomes
MGM101	-0.9	mitochondrial nucleoid protein
DAD2	-1.3	outer kinetochore protein - part of Dam1 complex
TEN1	-2.3	protein involved in Telomeric pathways in association with Stn1
TRX1	-1.0	thioredoxin
VPS71	-0.9	component of the Swr1p complex that incorporates Htz1p into chromatin, required for vacuolar protein sorting
HUG1	-1.3	protein involved in the Mec1p-mediated checkpoint pathway that responds to DNA damage or replication arrest
MFT1	-1.2	mitochondrial targeting protein
TAF13	-1.0	TFIID subunit
IMP2	-1.0	protease
BUB2	-0.9	protein required for cell cycle arrest in response to loss of microtubule function
ABF2	-1.0	HMG-1 homolog
CIN4	-1.0	GTP-binding protein
TAF9	-1.2	TFIID subunit
SAP30	-1.7	subunit of the histone deacetylase B complex
JNM1	-1.1	coiled-coil domain protein required for proper nuclear migration during mitosis
YMR299C	-1.3	protein of unknown function; GFP-fusion protein localizes to the cytoplasm
SPO1	-0.9	similar to phospholipase B
HHF2	-2.3	histone H4
HHT2	-1.4	histone H3
NOP15	-1.4	ribosome biogenesis
GIM3	-1.4	bovine prefoldin subunit 4 homolog (putative)
KAR1	-1.0	involved in spindle pole body duplication and karyogamy
YNL213C	-1.2	questionable protein
NTG2	-1.1	endonuclease III DNA base excision repair N-glycosylase
HRT1	-1.1	Skp1-Cullin-F-box ubiquitin protein ligase (SCF) subunit
PSF3	-1.3	a subunit of the GINS complex required for chromosomal DNA replication
CIN5	-1.1	transcriptional activator
YNG1	-1.0	histone acetyltransferase complex component
SAS5	-1.0	involved in silencing at telomeres, HML and HMR
RFM1	-1.0	DNA-binding protein
SPS4	-1.6	sporulation-specific protein
SPC29	-1.3	spindle pole body component
SPP1	-0.9	compass (complex proteins associated with Set1p) component
MCM16	-1.8	outer kinetochore protein
DIB1	-1.3	protein required for mitosis
**Transcription**		
POP5	-1.3	RNase MRP subunit (putative); RNase P integral subunit
HTA2	-1.7	histone H2A
POP8	-1.5	RNase P integral subunit; subunit of RNase MRP (putative)
HAP3	-1.3	transcriptional activator protein of CYC1 (component of HAP2/HAP3 heteromer)
RRN10	-1.5	upstream activation factor subunit
LSM2	-1.2	snRNA-associated protein, Sm class
HHF1	-2.3	histone H4 (HHF1 and HHF2 code for identical proteins)
HHT1	-1.7	histone H3 (HHT1 and HHT2 code for identical proteins)
NRG2	-1.6	NRG1 homolog

Gene name	Fold change	Gene Product
NHP6B	-1.2	11 kDa nonhistone chromosomal protein
CBP6	-1.2	translational activator of COB mRNA
RPB5	-1.1	25 kDa RNA polymerase subunit (common to polymerases I, II and III)
POP7	-1.4	RNase MRP subunit (putative); RNase P integral subunit
NTC20	-1.4	splicing factor
SRB6	-2.1	part of Srb/Mediator complex; transcription factor
KRR1	-0.9	Involved in cell division and spore germination
HMLALPHA1	-1.6	mating type regulatory protein, silenced copy at HML
HMLALPHA2	-2.7	mating type regulatory protein, silenced copy at HML
SRD1	-1.2	protein involved in the processing of pre-rRNA to mature rRNA
PET18	-2.2	protein required for respiratory growth and stability of the mitochondrial genome
MATALPHA2	-3.0	mating type regulatory protein, expressed copy at MAT locus
MATALPHA1	-1.5	mating type regulatory protein, expressed copy at MAT locus
YCR087C-A	-1.2	questionable protein
HMRA1	-2.0	mating type regulatory protein, silenced copy at HMR locus
MSS2	-1.1	cox2 pre-mRNA splicing factor
CDC36	-1.1	basal transcription inhibitor; transcriptional regulator
NHP2	-2.2	HMG-like protein
NOP6	-1.8	protein with similarity to hydrophilins, which are involved in the adaptive response to hyperosmotic conditions
DTD1	-1.2	D-Tyr-tRNA(Tyr) deacylase
NRG1	-1.6	binds to UAS-1 in the STA1 promoter and can interact with Ssn6; transcriptional repressor
RPC11	-1.4	TFIIS-like small Pol III subunit C11
SNF11	-1.4	SWI/SNF global transcription activator complex component
TFB5	-1.9	Component of general transcription and DNA repair factor TFIIH
RRP8	-1.1	nucleolar protein required for efficient processing of pre-rRNA at site A2; methyltransferase homolog
RRP1	-1.0	involved in processing rRNA precursor species to mature rRNAs
CWC15	-2.0	Protein involved in mRNA splicing
TAF10	-1.2	TFIID subunit
HTA1	-1.7	histone H2A (HTA1 and HTA2 code for nearly identical proteins)
SRB7	-1.2	RNA polymerase II holoenzyme/mediator subunit
LSM6	-1.2	snRNP protein
NCB2	-1.6	Negative Cofactor B2 is the beta subunit of a negative regulator of RNA polymerase II holoenzyme
YDR412W	-0.9	protein required for cell viability
ADA2	-1.4	general transcriptional adaptor or co-activator
SMB1	-1.1	associated with U1 snRNP as part of the Sm-core that is common to all spliceosomal snRNPs
LSM4	-1.0	U6 snRNA associated protein
LSM5	-2.0	snRNP protein
BUR6	-2.0	transcriptional regulator
SMX2	-1.3	snRNP G protein (human Sm-G homolog)
LOC1	-2.0	nuclear protein involved in asymmetric localization of ASH1 mRNA
CGR1	-2.3	coiled-coil protein
RPB9	-1.3	RNA polymerase II core subunit
POP6	-1.4	RNase P integral subunit; subunit of RNase MRP (putative)
SPT4	-3.1	transcription elongation protein
SMD1	-1.1	homolog of human core snRNP protein D1, involved in snRNA maturation
PRP38	-1.1	RNA splicing factor
RRP46	-1.2	3'->5' exoribonuclease
SYF2	-0.9	SYnthetic lethal with CDC40
CPD1	-1.9	2',3'-cyclic nucleotide 3'-phosphodiesterase
YGR251W	-1.2	protein required for cell viability

Gene name	Fold change	Gene Product
PXR1	-1.3	possible telomerase regulator or RNA-binding protein
SNF6	-0.9	chromatin remodeling Snf/Swi complex subunit
BCD1	-0.9	box C/D snoRNA accumulation
SRB2	-1.5	RNA polymerase II holoenzyme/mediator subunit
NOP10	-1.3	H/ACA-box snoRNPs component
LRP1	-1.6	like rRNA Processing protein involved in regulation of DNA repair and recombination
YHR085W	-1.2	protein of unknown function, essential for viability, may be involved in rRNA processing
GAR1	-1.4	small nucleolar RNP protein
RPC10	-1.6	RNA polymerase II subunit
IMP3	-0.9	U3 snoRNP protein
IST3	-2.1	U2 snRNP associated protein
MSL1	-1.2	U2 snRNP component
YAP5	-1.5	transcription factor
RPC17	-1.2	RNA polymerase III subunit C17
TAD2	-0.9	tRNA-specific adenosine deaminase subunit
ASF1	-1.1	anti-silencing protein that causes depression of silent loci when overexpressed
RPB4	-1.0	RNA polymerase II fourth largest subunit
RPA34	-1.5	RNA polymerase I subunit A34.5
LSM8	-2.1	snRNP protein
RPA12	-1.0	RNA polymerase I A12.2 subunit
YJR141W	-1.3	protein required for cell viability
MRT4	-1.3	mRna turnover 4
UTP11	-1.0	U3 snoRNP protein
YKR022C	-1.3	protein required for cell viability
YLR016C	-1.3	questionable protein
YLR022C	-1.0	protein required for cell viability
FYV7	-2.3	protein involved in processing the 35S rRNA primary transcript to generate the 20S and 27SA2 pre-rRNA transcripts
YLR145W	-2.2	protein required for cell viability
SMD3	-1.1	core snRNP protein
PUS5	-1.8	RNA:pseudouridine (Psi)-synthase
PDR8	-1.1	zinc finger transcription factor
SMD2	-0.9	U1 snRNP protein of the Sm class
YHC1	-1.7	U1 snRNP protein required for pre-mRNA splicing
TAD3	-0.9	tRNA-specific adenosine deaminase subunit
YLR405W	-0.9	dihydrouridine synthase 4
TSR2	-1.3	twenty S rRNA accumulation
LSM3	-2.5	snRNP protein
TRM9	-0.9	mcm5U/mcm5s2U tRNA carboxyl methyltransferase
MFT1	-1.2	mitochondrial targeting protein
TAF13	-1.0	TFIID subunit
YML107C	-1.3	protein of unknown function; GFP-fusion protein localizes to the nuclear periphery
DAT1	-1.2	datin, oligo(dA)/oligo(dT)-binding protein
SEN15	-1.2	tetrameric tRNA splicing endonuclease 15kDa subunit
CTL1	-1.3	RNA triphosphatase
TAF9	-1.2	TFIID subunit
SAP30	-1.7	subunit of the histone deacetylase B complex
RLP7	-1.3	nucleolar protein related to ribosomal protein L7
SPO1	-0.9	similar to phospholipase B
HHF2	-2.3	histone H4
HHT2	-1.4	histone H3
RPC19	-1.2	RNA polymerases I (A) and III (C) subunit
LSM7	-1.6	snRNP protein

Gene name	Fold change	Gene Product
RPB11	-1.2	RNA polymerase II core subunit
YOL101C	-0.9	questionable protein
INO4	-1.7	basic helix-loop-helix (bHLH) protein
HRT1	-1.1	Skp1-Cullin-F-box ubiquitin protein ligase (SCF) subunit
DCP1	-0.9	mRNA decapping enzyme
YOR004W	-1.2	protein required for cell viability
CIN5	-1.1	transcriptional activator
BUD21	-1.5	U3 snoRNP protein
SPP2	-1.7	required for final stages of splicesome maturation
SME1	-1.0	spliceosomal snRNA-associated Sm core protein required for mRNA splicing, also likely associated with telomerase TLC1 RNA
RPB10	-1.9	RNA polymerase II core subunit
SAS5	-1.0	involved in silencing at telomeres, HML and HMR
RPB8	-0.9	DNA-directed RNA polymerase I, II, III 16 KD subunit
YOR287C	-1.7	protein required for cell viability
RRS1	-1.2	regulator for ribosome synthesis
MBF1	-1.9	multiprotein bridging factor
MET31	-1.2	transcriptional regulator of sulfur amino acid metabolism
YPL047W	-1.4	questionable protein
SPP1	-0.9	compass (complex proteins associated with Set1p) component
TIF6	-1.0	translation initiation factor 6 (eIF6)
BRR1	-0.9	spliceosomal snRNP component
DIB1	-1.3	protein required for mitosis
RDS3	-2.0	protein required for cell viability
SNT309	-1.5	protein complex component associated with the splicing factor Prp19p
YPR143W	-1.3	protein required for cell viability
RPO26	-1.5	RNA polymerases I, II, and III subunit
**Protein synthesis**		
ECM1	-1.7	putative transmembrane domain protein involved in cell wall biogenesis
CBP6	-1.2	translational activator of COB mRNA
MRPL37	-1.3	mitochondrial ribosomal protein of the large subunit
MRPL27	-1.7	mitochondrial ribosomal protein of the large subunit
KRR1	-0.9	Involved in cell division and spore germination
MRPL32	-1.7	ribosomal protein (YmL32)
IMG2	-1.1	mitochondrial ribosomal protein of the small subunit
DTD1	-1.2	D-Tyr-tRNA(Tyr) deacylase
YDR115W	-1.4	questionable protein
YDR185C	-1.1	questionable protein
RPL27B	-0.9	ribosomal protein L27B
SLF1	-1.1	La motif-containing proteins that modulate mRNA translation
NOP16	-1.4	ribosome biogenesis
RSM18	-1.1	mitochondrial ribosome small subunit component
RPS24A	-1.0	ribosomal protein S24A
RPL23B	-0.9	ribosomal protein L23B (L17aB) (YL32)
RPL22B	-1.6	ribosomal protein L22B (L1c) (rp4) (YL31)
LOC1	-2.0	nuclear protein involved in asymmetric localization of ASH1 mRNA
RPL29	-0.9	ribosomal protein L29 (YL43)
CGR1	-2.3	coiled-coil protein
MRPL25	-1.3	mitochondrial ribosomal protein of the large subunit
YGR201C	-1.0	questionable protein
RSM27	-2.0	mitochondrial ribosome small subunit component
RPL27A	-1.0	ribosomal protein L27A
IMP3	-0.9	U3 snoRNP protein
PET130	-1.1	protein required for respiratory growth
MRPL49	-0.9	mitochondrial ribosomal protein of the large subunit

Gene name	Fold change	Gene Product
RPS21B	-1.7	ribosomal protein S21B (S26B) (YS25)
RPL39	-1.3	ribosomal protein L39 (L46) (YL40)
RPL43B	-1.0	ribosomal protein L43B
MRP17	-1.5	ribosomal protein MRP17
MRT4	-1.3	mRna turnover 4
MRPL31	-1.0	mitochondrial ribosomal protein of the large subunit
RPS27A	-1.0	ribosomal protein S27A (rp61) (YS20)
RPS21A	-1.2	ribosomal protein S21A (S26A) (YS25)
RPL37A	-1.3	ribosomal protein L37A (L43) (YL35)
YLR221C	-0.9	riboSome Assembly
RPS28B	-1.0	ribosomal protein S28B (S33B) (YS27)
RPS30A	-1.1	ribosomal protein S30A
MRPL15	-1.4	mitochondrial ribosomal protein of the large subunit
RPL38	-1.0	ribosomal protein L38
RPS29A	-1.1	ribosomal protein S29A (S36A) (YS29)
MRPL39	-1.2	mitochondrial ribosomal protein of the large subunit
TRM9	-0.9	mcm5U/mcm5s2U tRNA carboxyl methyltransferase
RPS18B	-0.9	ribosomal protein S18B
MRPS8	-1.2	mitochondrial ribosomal protein of the small subunit
MRPS17	-0.9	mitochondrial ribosomal protein of the small subunit
MRPL44	-1.3	mitochondrial ribosomal protein of the large subunit
TIF11	-1.2	translation initiation factor eIF1A
MRPL33	-1.6	mitochondrial ribosomal protein of the large subunit
RLP7	-1.3	nucleolar protein related to ribosomal protein L7
NOP15	-1.4	ribosome biogenesis
SUI1	-1.7	translation initiation factor eIF1
MRPL50	-1.2	mitochondrial ribosomal protein of the large subunit, not essential for mitochondrial translation
RSM19	-1.0	mitochondrial ribosome small subunit component
RPS28A	-1.3	ribosomal protein S28A (S33A) (YS27)
RPS30B	-1.3	ribosomal protein S30B
RPL33B	-1.2	ribosomal protein L33B (L37B) (rp47) (YL37)
RRS1	-1.2	regulator for ribosome synthesis
TIF6	-1.0	translation initiation factor 6 (eIF6)
MRPL51	-1.1	mitochondrial ribosomal protein of the large subunit
YPR143W	-1.3	protein required for cell viability
MRP2	-0.9	mitochondrial ribosomal protein, small subunit
**Protein fate**		
PEX22	-1.6	product of gene unknown
LDB7	-0.9	protein involved in mannosylphosphorylation of cell wall mannoproteins
PRE7	-0.9	proteasome subunit
KTI11	-1.2	similar to Hemiascomycetous yeast protein
RER2	-0.9	cis-prenyltransferase
MRS5	-1.0	involved in mitochondrial biogenesis
ATG14	-1.5	required for autophagy
YSY6	-3.4	protein that participates in secretory pathway
UBS1	-1.0	general positive regulator of CDC34
DER1	-1.1	endoplasmic reticulum membrane protein, required for the protein degradation process associated with the ER, involved in the retrograde transport of misfolded or unassembled proteins
ATG12	-2.3	component of the autophagic system
SHG1	-1.5	compass (complex proteins associated with Set1p) component
MAK31	-1.8	member of the Sm protein family
YCR060W	-1.5	questionable protein
APC11	-1.2	anaphase promoting complex (APC) subunit
PEX19	-1.2	40 kDa farnesylated protein associated with peroxisomes

Gene name	Fold change	Gene Product
COX9	-2.0	cytochrome c oxidase subunit VIIa
SRP14	-1.4	signal recognition particle subunit
MSS2	-1.1	cox2 pre-mRNA splicing factor
OST4	-0.9	oligosaccharyltransferase subunit
TFB5	-1.9	Component of general transcription and DNA repair factor TFIIH
SSS1	-1.6	ER protein-translocase complex subunit
RUB1	-1.3	ubiquitin-like protein
TAF10	-1.2	TFIID subunit
UBC1	-1.2	ubiquitin-conjugating enzyme
CSN9	-2.7	COP9 signalosome (CSN) subunit
YDR185C	-1.1	questionable protein
GPI11	-1.3	protein involved in glycosylphosphatidylinositol (GPI) biosynthesis
TIM11	-1.7	mitochondrial F1F0-ATPase subunit e
SEM1	-2.6	Regulator of exocytosis and pseudohyphal differentiation
ADA2	-1.4	general transcriptional adaptor or co-activator
YDR459C	-1.2	likely functions in pathway(s) outside Ras
FPR2	-1.0	FKBP13 (FK506 binding protein)|peptidyl-prolyl cis-trans isomerase (PPIase)
QCR7	-1.1	ubiquinol cytochrome C oxidoreductase subunit 7
GIM4	-2.0	bovine prefoldin subunit 2 homolog (putative)
UBC8	-1.0	ubiquitin-conjugating enzyme; ubiquitin-protein ligase
TIM9	-1.8	essential subunit of the TIM22-complex for mitochondrial protein import
SBH2	-1.5	Sbh1p homolog
PET117	-1.5	protein required for assembly of cytochrome c oxidase
YPT1	-0.9	GTP-binding protein; ras homolog
YPI1	-1.0	PP1 phosphatase inhibitor
LYS5	-1.0	alpha aminoadipate reductase phosphopantetheinyl transferase
BRR6	-1.5	nuclear envelope protein
SMD1	-1.1	homolog of human core snRNP protein D1, involved in snRNA maturation
VMA21	-1.7	protein involved in vacuolar H-ATPase assembly or function
TRX2	-1.0	thioredoxin
PEX21	-1.3	peroxin
MRS11	-1.4	subunit of the Tim22-complex
VMA22	-1.9	required for V-ATPase activity
WSS1	-1.6	weak suppressor of smt3
SSP1	-1.2	essential for meiosis and spore formation
URM1	-1.5	ubiquitin-like protein
YRB2	-0.9	nuclear protein that interacts with Gsp1p and Crm1p
FMC1	-1.2	assembly factor of ATP synthase in heat stress
IST3	-2.1	U2 snRNP associated protein
MSL1	-1.2	U2 snRNP component
STS1	-1.1	restores protein transport when overexpressed and rRNA stability to a sec23 mutation
YJL104W	-0.9	presequence translocase-asssociated motor, constituent of the mitochondrial import motor PAM
PFD1	-1.3	bovine prefoldin subunit 1 homolog (putative)
GON7	-1.4	protein proposed to be involved in the transfer of mannosylphosphate groups onto N-linked oligosaccharides
SPC1	-2.5	homolog of the SPC12 subunit of mammalian signal peptidase complex
APS2	-1.0	clathrin associated protein complex small subunit
YJR097W	-1.5	protein that may function as a cochaperone
TIM8	-1.0	mitochondrial protein import machinery subunit
VPS24	-1.3	involved in secretion
VPH2	-1.0	25.2 kDa protein involved in assembly of vacuolar H(+) ATPase
SRP21	-1.7	signal recognition particle component
DID2	-1.2	class E vacuolar-protein sorting and endocytosis factor

Gene name	Fold change	Gene Product
COX17	-1.5	cysteine-rich protein
ATG10	-1.5	protein-conjugating enzyme
COX12	-1.1	cytochrome c oxidase subunit VIb
TRX1	-1.0	thioredoxin
SRN2	-1.1	suppressor of rna1-1 mutation
APS1	-1.0	clathrin associated protein complex small subunit
YKE2	-1.1	Gim complex component
YLR243W	-1.1	protein required for cell viability
UBC12	-1.7	ubiquitin-conjugating enzyme
VPS71	-0.9	component of the Swr1p complex that incorporates Htz1p into chromatin, required for vacuolar protein sorting
MFT1	-1.2	mitochondrial targeting protein
COX14	-1.2	mitochondrial membrane protein
IMP2	-1.0	protease
TAF9	-1.2	TFIID subunit
COX7	-2.2	cytochrome c oxidase subunit VII
SAP30	-1.7	subunit of the histone deacetylase B complex
GOT1	-1.2	membrane protein
GPI15	-1.6	human Pig-H homolog
TOM7	-0.9	translocase of the outer mitochondrial membrane
YPT53	-1.0	GTP-binding protein; rab family
GIM3	-1.4	bovine prefoldin subunit 4 homolog (putative)
ATX1	-1.4	copper binding homeostasis protein (putative)
HUB1	-1.5	ubiquitin-like modifier
HRT1	-1.1	Skp1-Cullin-F-box ubiquitin protein ligase (SCF) subunit
YSP3	-1.0	subtilisin-like protease III
HSP10	-1.6	heat shock protein 10
PEP12	-1.0	syntaxin (T-SNARE), vacuolar
TOM6	-2.1	mitochondrial outer membrane import receptor subunit, 6 kD
YNG1	-1.0	histone acetyltransferase complex component
ARF3	-1.1	GTP-binding ADP-ribosylation factor
OST2	-1.4	oligosaccharyltransferase epsilon subunit
SAS5	-1.0	involved in silencing at telomeres, HML and HMR
MGE1	-0.9	GrpE homolog
NAT5	-2.4	N-acetyltransferase
ARL3	-0.9	ADP-ribosylation factor-like protein, member of the arf-sar family in the ras superfamily
VPS28	-1.0	protein involved in vacuolar traffic
SPP1	-0.9	compass (complex proteins associated with Set1p) component
PPT2	-2.1	phosphopantetheine:protein transferase (PPTase)
ATG5	-1.6	Involved in autophagy
TFP3	-1.1	vacuolar ATPase V0 domain subunit c' (17 kDa)|vacuolar H(+) ATPase 17 kDa subunit C
MAK3	-1.1	N-acetyltransferase
BRR1	-0.9	spliceosomal snRNP component
**Regulation of metabolism and protein function**		
NRG2	-1.6	NRG1 homolog
POL30	-1.1	proliferating Cell Nuclear Antigen (PCNA)
HMLALPHA1	-1.6	mating type regulatory protein, silenced copy at HML
HMLALPHA2	-2.7	mating type regulatory protein, silenced copy at HML
MATALPHA2	-3.0	mating type regulatory protein, expressed copy at MAT locus
MATALPHA1	-1.5	mating type regulatory protein, expressed copy at MAT locus
HMRA1	-2.0	mating type regulatory protein, silenced copy at HMR locus
INH1	-1.4	ATPase inhibitor
NCB2	-1.6	Negative Cofactor B2 is the beta subunit of a negative regulator of RNA polymerase II holoenzyme

Gene name	Fold change	Gene Product
ADA2	-1.4	general transcriptional adaptor or co-activator
BUR6	-2.0	transcriptional regulator
YPI1	-1.0	PP1 phosphatase inhibitor
PEX21	-1.3	peroxin
YNG2	-1.2	NuA4 histone acetyltransferase complex component
SPL2	-1.7	suppressor of plc1-delta
KTI12	-1.1	elongator associated protein
BUB2	-0.9	protein required for cell cycle arrest in response to loss of microtubule function
MBF1	-1.9	multiprotein bridging factor
ERI1	-1.2	ER-associated Ras Inhibitor
**Cellular transport, transport facilities and transport routes**		
PEX22	-1.6	product of gene unknown
YBR043C	-1.0	protein of unknown function, may have a role in drug resistance
MRS5	-1.0	involved in mitochondrial biogenesis
YMC2	-1.0	mitochondrial carrier protein
YSY6	-3.4	protein that participates in secretory pathway
UBS1	-1.0	general positive regulator of CDC34
ATG12	-2.3	component of the autophagic system
TRS20	-1.9	trapp subunit of 20 kDa
YPT10	-1.0	similar to Rab proteins and other small GTP-binding proteins
CTP1	-1.2	citrate tranporter
GRX1	-1.1	glutaredoxin
TRX3	-0.9	thioredoxin
PEX19	-1.2	40 kDa farnesylated protein associated with peroxisomes
COX9	-2.0	cytochrome c oxidase subunit VIIa
SRP14	-1.4	signal recognition particle subunit
MSS2	-1.1	cox2 pre-mRNA splicing factor
INH1	-1.4	ATPase inhibitor
SSS1	-1.6	ER protein-translocase complex subunit
UBC1	-1.2	ubiquitin-conjugating enzyme
TIM11	-1.7	mitochondrial F1F0-ATPase subunit e
SEM1	-2.6	Regulator of exocytosis and pseudohyphal differentiation
DYN2	-2.1	dynein light chain (putative)
TIM9	-1.8	essential subunit of the TIM22-complex for mitochondrial protein import
SBH2	-1.5	Sbh1p homolog
YPT1	-0.9	GTP-binding protein; ras homolog
LOC1	-2.0	nuclear protein involved in asymmetric localization of ASH1 mRNA
ERV14	-0.9	14 kDa protein found on ER-derived vesicles
BRR6	-1.5	nuclear envelope protein
VMA7	-1.2	vacuolar ATPase V1 domain subunit F (14 kDa)
LST7	-1.1	required for amino acid permease transport from the Golgi to the cell surface
YGR131W	-1.7	questionable protein
TRX2	-1.0	thioredoxin
PEX21	-1.3	peroxin
MRS11	-1.4	subunit of the Tim22-complex
VMA10	-1.2	vacuolar ATPase V1 domain subunit G (13 kDa)
BET1	-1.6	membrane protein required for vesicular transport between the endoplasmic reticulum and Golgi complex
YIL040W	-1.0	protein of unknown function, localizes to the endoplasmic reticulum
YRB2	-0.9	nuclear protein that interacts with Gsp1p and Crm1p
QDR1	-0.9	multidrug resistance transporter
FLX1	-0.9	FAD carrier protein
STS1	-1.1	restores protein transport when overexpressed and rRNA stability to a sec23 mutation

Gene name	Fold change	Gene Product
SYS1	-1.0	multicopy suppressor of ypt6 null mutation
YJL104W	-0.9	presequence translocase-asssociated motor, constituent of the mitochondrial import motor PAM
NCE101	-1.4	involved in secretion of proteins that lack classical secretory signal sequences
CYC1	-0.9	iso-1-cytochrome c
APS2	-1.0	clathrin associated protein complex small subunit
TIM8	-1.0	mitochondrial protein import machinery subunit
SFT1	-1.5	v-SNARE
VPS24	-1.3	involved in secretion
SRP21	-1.7	signal recognition particle component
MTR2	-1.8	mRNA transport regulator
DID2	-1.2	class E vacuolar-protein sorting and endocytosis factor
BET3	-0.9	transport protein particle (TRAPP) component
COX17	-1.5	cysteine-rich protein
ATG10	-1.5	protein-conjugating enzyme
YLR004C	-1.0	questionable protein
YLR016C	-1.3	questionable protein
COX12	-1.1	cytochrome c oxidase subunit VIb
TRX1	-1.0	thioredoxin
SRN2	-1.1	suppressor of rna1-1 mutation
ZRT2	-0.9	low affinity zinc transport protein
APS1	-1.0	clathrin associated protein complex small subunit
ARV1	-1.0	protein involved in sterol distribution
COX8	-1.0	cytochrome c oxidase chain VIII
ERV25	-1.0	vesicle coat component
VPS71	-0.9	component of the Swr1p complex that incorporates Htz1p into chromatin, required for vacuolar protein sorting
MFT1	-1.2	mitochondrial targeting protein
ATP18	-1.7	ATP synthase associated protein
YML107C	-1.3	protein of unknown function; GFP-fusion protein localizes to the nuclear periphery
VPS20	-0.9	vaculolar protein sorting (putative)
GFD1	-1.2	protein of the nuclear pore complex
COX7	-2.2	cytochrome c oxidase subunit VII
GOT1	-1.2	membrane protein
JNM1	-1.1	coiled-coil domain protein required for proper nuclear migration during mitosis
YMR299C	-1.3	protein of unknown function; GFP-fusion protein localizes to the cytoplasm
NCE103	-1.4	carbonic anhydrase-like protein
TOM7	-0.9	translocase of the outer mitochondrial membrane
YPT53	-1.0	GTP-binding protein; rab family
CYB5	-1.1	cytochrome b5
ATX1	-1.4	copper binding homeostasis protein (putative)
ATP19	-1.2	ATP synthase subunit k homolog
PEP12	-1.0	syntaxin (T-SNARE), vacuolar
TOM6	-2.1	mitochondrial outer membrane import receptor subunit, 6 kD
ARF3	-1.1	GTP-binding ADP-ribosylation factor
ORT1	-1.0	Mitochondrial ornithine carrier - member of the mitochondrial carrier family
MGE1	-0.9	GrpE homolog
RET3	-0.9	vesicle coat component
ARL3	-0.9	ADP-ribosylation factor-like protein, member of the arf-sar family in the ras superfamily
VPS28	-1.0	protein involved in vacuolar traffic
PPT2	-2.1	phosphopantetheine:protein transferase (PPTase)
ATG5	-1.6	Involved in autophagy
OYE3	-1.8	NADPH dehydrogenase

Gene name	Fold change	Gene Product
TFP3	-1.1	vacuolar ATPase V0 domain subunit c' (17 kDa)|vacuolar H(+) ATPase 17 kDa subunit C
ATP15	-1.3	ATP synthase epsilon subunit
ATP20	-1.5	ATP synthase subunit g homolog
**Cellular communication /Signal transduction mechanism**		
MFA1	-2.0	a-factor mating pheromone precursor
STE18	-1.6	G protein gamma subunit; coupled to mating factor receptor
MFA2	-1.7	a-factor mating pheromone precursor
ZEO1	-2.1	Mid2p-interacting protein, modulates the PKC1-MPK1 cell integrity pathway
ERI1	-1.2	ER-associated Ras Inhibitor
**Cell rescue, defense and virulence**		
YBR043C	-1.0	protein of unknown function, may have a role in drug resistance
GPX2	-1.4	phospholipid hydroperoxide glutathione peroxidase
GRX1	-1.1	glutaredoxin
YCR060W	-1.5	questionable protein
TRX3	-0.9	thioredoxin
NOP6	-1.8	protein with similarity to hydrophilins, which are involved in the adaptive response to hyperosmotic conditions
RAD57	-1.2	RecA homolog; interacts with Rad 55p
NRG1	-1.6	binds to UAS-1 in the STA1 promoter and can interact with Ssn6; transcriptional repressor
PET100	-1.5	cytochrome c oxidase-specific assembly factor
CTA1	-1.8	catalase A
TSA2	-1.0	thioredoxin-peroxidase
FPR2	-1.0	FKBP13 (FK506 binding protein)|peptidyl-prolyl cis-trans isomerase (PPIase)
TIM9	-1.8	essential subunit of the TIM22-complex for mitochondrial protein import
YER187W	-2.1	questionable protein
SNO3	-0.9	protein is related with B(1) biosynthesis during the exponential phase
TRX2	-1.0	thioredoxin
MRS11	-1.4	subunit of the Tim22-complex
DOG2	-0.9	2-deoxyglucose-6-phosphate phosphatase
CUP1-1	-2.0	copper binding metallothionein
CUP1-2	-2.1	copper binding metallothionein
VMA22	-1.9	required for V-ATPase activity
LRP1	-1.6	like rRNA Processing protein involved in regulation of DNA repair and recombination
SPL2	-1.7	suppressor of plc1-delta
EST3	-1.8	Subunit of the telomerase
QDR1	-0.9	multidrug resistance transporter
ASF1	-1.1	anti-silencing protein that causes depression of silent loci when overexpressed
RPB4	-1.0	RNA polymerase II fourth largest subunit
PFD1	-1.3	bovine prefoldin subunit 1 homolog (putative)
GON7	-1.4	protein proposed to be involved in the transfer of mannosylphosphate groups onto N-linked oligosaccharides
KTI12	-1.1	elongator associated protein
YKR049C	-1.0	protein localized to the mitochondria
RPS21A	-1.2	ribosomal protein S21A (S26A) (YS25)
TRX1	-1.0	thioredoxin
ASP3-1	-1.2	nitrogen catabolite-regulated cell-wall L-asparaginase II
ASP3-2	-1.0	nitrogen catabolite-regulated cell-wall L-asparaginase II
ASP3-3	-1.0	nitrogen catabolite-regulated cell-wall L-asparaginase II
ASP3-4	-1.2	nitrogen catabolite-regulated cell-wall L-asparaginase II

Gene name	Fold change	Gene Product
PDR8	-1.1	zinc finger transcription factor
TRM9	-0.9	mcm5U/mcm5s2U tRNA carboxyl methyltransferase
HUG1	-1.3	protein involved in the Mec1p-mediated checkpoint pathway that responds to DNA damage or replication arrest
SIP18	-1.1	salt-Induced Protein
HOR7	-1.2	hyperosmolarity-responsive gene
ATX1	-1.4	copper binding homeostasis protein (putative)
ZEO1	-2.1	Mid2p-interacting protein, modulates the PKC1-MPK1 cell integrity pathway
HSP10	-1.6	heat shock protein 10
CIN5	-1.1	transcriptional activator
MGE1	-0.9	GrpE homolog
RFM1	-1.0	DNA-binding protein
ARR2	-1.9	arsenate reductase
**Interaction with the environment**		
YBL028C	-2.1	questionable protein
SRB6	-2.1	part of Srb/Mediator complex; transcription factor
HMLALPHA1	-1.6	mating type regulatory protein, silenced copy at HML
HMLALPHA2	-2.7	mating type regulatory protein, silenced copy at HML
MATALPHA2	-3.0	mating type regulatory protein, expressed copy at MAT locus
MATALPHA1	-1.5	mating type regulatory protein, expressed copy at MAT locus
HMRA2	-3.0	mating type regulatory protein, silenced copy at HMR locus
HMRA1	-2.0	mating type regulatory protein, silenced copy at HMR locus
CDC36	-1.1	basal transcription inhibitor; transcriptional regulator
RAD57	-1.2	RecA homolog; interacts with Rad 55p
NRG1	-1.6	binds to UAS-1 in the STA1 promoter and can interact with Ssn6; transcriptional repressor
SNF11	-1.4	SWI/SNF global transcription activator complex component
CSN9	-2.7	COP9 signalosome (CSN) subunit
MFA1	-2.0	a-factor mating pheromone precursor
SLF1	-1.1	La motif-containing proteins that modulate mRNA translation
FAR7	-1.0	protein involved in G1 cell cycle arrest in response to pheromone, in a pathway different from the Far1p-dependent pathway
AGA2	-1.4	a-agglutinin adhesion subunit
VMA7	-1.2	vacuolar ATPase V1 domain subunit F (14 kDa)
SNF6	-0.9	chromatin remodeling Snf/Swi complex subunit
VMA10	-1.2	vacuolar ATPase V1 domain subunit G (13 kDa)
VMA22	-1.9	required for V-ATPase activity
SPL2	-1.7	suppressor of plc1-delta
STE18	-1.6	G protein gamma subunit; coupled to mating factor receptor
VPH2	-1.0	25.2 kDa protein involved in assembly of vacuolar H(+) ATPase
COX17	-1.5	cysteine-rich protein
ZRT2	-0.9	low affinity zinc transport protein
KAR5	-1.1	coiled-coil membrane protein
SAP30	-1.7	subunit of the histone deacetylase B complex
MFA2	-1.7	a-factor mating pheromone precursor
KAR1	-1.0	involved in spindle pole body duplication and karyogamy
ATX1	-1.4	copper binding homeostasis protein (putative)
HUB1	-1.5	ubiquitin-like modifier
YOL101C	-0.9	questionable protein
CIN5	-1.1	transcriptional activator
ISU1	-1.1	iron-sulfur cluster nifU-like protein
TFP3	-1.1	vacuolar ATPase V0 domain subunit c' (17 kDa)|vacuolar H(+) ATPase 17 kDa subunit C
ATP15	-1.3	ATP synthase epsilon subunit
RDS3	-2.0	protein required for cell viability

Gene name	Fold change	Gene Product
**Transposable elements, viral and plasmid proteins**		
MAK31	-1.8	member of the Sm protein family
RTT102	-0.9	regulator of Ty1 Transposition
**Cell fate**		
NHP6B	-1.2	11 kDa nonhistone chromosomal protein
SPR6	-1.3	sporulation-specific protein
ASF1	-1.1	anti-silencing protein that causes depression of silent loci when overexpressed
YKL082C	-0.9	required for normal pre-rRNA Processing
KTI12	-1.1	elongator associated protein
DAD2	-1.3	outer kinetochore protein - part of Dam1 complex
BUB2	-0.9	protein required for cell cycle arrest in response to loss of microtubule function
SAP30	-1.7	subunit of the histone deacetylase B complex
YMR269W	-2.2	protein possibly involved in protein synthesis
HUB1	-1.5	ubiquitin-like modifier
**Development (Systemic)**		
HMLALPHA1	-1.6	mating type regulatory protein, silenced copy at HML
HMLALPHA2	-2.7	mating type regulatory protein, silenced copy at HML
MATALPHA2	-3.0	mating type regulatory protein, expressed copy at MAT locus
MATALPHA1	-1.5	mating type regulatory protein, expressed copy at MAT locus
HMRA1	-2.0	mating type regulatory protein, silenced copy at HMR locus
AGA2	-1.4	a-agglutinin adhesion subunit
KAR5	-1.1	coiled-coil membrane protein
KAR1	-1.0	involved in spindle pole body duplication and karyogamy
HUB1	-1.5	ubiquitin-like modifier
**Biogenesis of cellular components**		
ACS1	-1.5	acetyl CoA synthetase
ECM1	-1.7	putative transmembrane domain protein involved in cell wall biogenesis
LDB7	-0.9	protein involved in mannosylphosphorylation of cell wall mannoproteins
RER2	-0.9	cis-prenyltransferase
MRS5	-1.0	involved in mitochondrial biogenesis
ATG12	-2.3	component of the autophagic system
SHG1	-1.5	compass (complex proteins associated with Set1p) component
YPT10	-1.0	similar to Rab proteins and other small GTP-binding proteins
MRPL37	-1.3	mitochondrial ribosomal protein of the large subunit
MRPL27	-1.7	mitochondrial ribosomal protein of the large subunit
CTP1	-1.2	citrate tranporter
MRPL32	-1.7	ribosomal protein (YmL32)
PET18	-2.2	protein required for respiratory growth and stability of the mitochondrial genome
IMG2	-1.1	mitochondrial ribosomal protein of the small subunit
PEX19	-1.2	40 kDa farnesylated protein associated with peroxisomes
RAD57	-1.2	RecA homolog; interacts with Rad 55p
DAD1	-1.7	Duo1 and Dam1 interacting; localized to intranuclear spindles and spindle pole bodies
STN1	-1.2	involved in telomere length regulation
YDR115W	-1.4	questionable protein
TAF10	-1.2	TFIID subunit
SPC19	-1.2	spindle pole component
DYN2	-2.1	dynein light chain (putative)
ADA2	-1.4	general transcriptional adaptor or co-activator
GIM4	-2.0	bovine prefoldin subunit 2 homolog (putative)

Gene name	Fold change	Gene Product
TIM9	-1.8	essential subunit of the TIM22-complex for mitochondrial protein import
BIM1	-2.0	binding to microtubules
RSM18	-1.1	mitochondrial ribosome small subunit component
SPR6	-1.3	sporulation-specific protein
MDM39	-1.2	spore wall formation. Mitochondrial distribution and morphology
CGR1	-2.3	coiled-coil protein
BRR6	-1.5	nuclear envelope protein
MRPL25	-1.3	mitochondrial ribosomal protein of the large subunit
TRX2	-1.0	thioredoxin
RSM27	-2.0	mitochondrial ribosome small subunit component
PEX21	-1.3	peroxin
MRS11	-1.4	subunit of the Tim22-complex
EST3	-1.8	Subunit of the telomerase
YRB2	-0.9	nuclear protein that interacts with Gsp1p and Crm1p
SYS1	-1.0	multicopy suppressor of ypt6 null mutation
MRPL49	-0.9	mitochondrial ribosomal protein of the large subunit
YJL104W	-0.9	presequence translocase-asssociated motor, constituent of the mitochondrial import motor PAM
RPA34	-1.5	RNA polymerase I subunit A34.5
PFD1	-1.3	bovine prefoldin subunit 1 homolog (putative)
GON7	-1.4	protein proposed to be involved in the transfer of mannosylphosphate groups onto N-linked oligosaccharides
ABM1	-2.0	protein of unknown function, required for normal microtubule organization
MRP17	-1.5	ribosomal protein MRP17
YKL082C	-0.9	required for normal pre-rRNA Processing
MRPL31	-1.0	mitochondrial ribosomal protein of the large subunit
DAD2	-1.3	outer kinetochore protein - part of Dam1 complex
TEN1	-2.3	protein involved in Telomeric pathways in association with Stn1
TRX1	-1.0	thioredoxin
YKE2	-1.1	Gim complex component
MRPL15	-1.4	mitochondrial ribosomal protein of the large subunit
MRPL39	-1.2	mitochondrial ribosomal protein of the large subunit
BUB2	-0.9	protein required for cell cycle arrest in response to loss of microtubule function
KAR5	-1.1	coiled-coil membrane protein
ABF2	-1.0	HMG-1 homolog
CIN4	-1.0	GTP-binding protein
MRPS8	-1.2	mitochondrial ribosomal protein of the small subunit
MRPS17	-0.9	mitochondrial ribosomal protein of the small subunit
MRPL44	-1.3	mitochondrial ribosomal protein of the large subunit
TAF9	-1.2	TFIID subunit
MRPL33	-1.6	mitochondrial ribosomal protein of the large subunit
JNM1	-1.1	coiled-coil domain protein required for proper nuclear migration during mitosis
YMR299C	-1.3	protein of unknown function; GFP-fusion protein localizes to the cytoplasm
GIM3	-1.4	bovine prefoldin subunit 4 homolog (putative)
KAR1	-1.0	involved in spindle pole body duplication and karyogamy
YNL213C	-1.2	questionable protein
MRPL50	-1.2	mitochondrial ribosomal protein of the large subunit, not essential for mitochondrial translation
RSM19	-1.0	mitochondrial ribosome small subunit component
MDM12	-1.2	mitochondrial outer membrane protein
ZEO1	-2.1	Mid2p-interacting protein, modulates the PKC1-MPK1 cell integrity pathway
HSP10	-1.6	heat shock protein 10
YNG1	-1.0	histone acetyltransferase complex component
SPC29	-1.3	spindle pole body component

Gene name	Fold change	Gene Product
SPP1	-0.9	compass (complex proteins associated with Set1p) component
ATG5	-1.6	Involved in autophagy
YPL250C	-1.3	Protein that interacts with the cytoskeleton and is involved in chromatin organization and nuclear transport
MRPL51	-1.1	mitochondrial ribosomal protein of the large subunit
MRP2	-0.9	mitochondrial ribosomal protein, small subunit
**Cell type differentiation**		
NRG2	-1.6	NRG1 homolog
NHP6B	-1.2	11 kDa nonhistone chromosomal protein
SRB6	-2.1	part of Srb/Mediator complex; transcription factor
BUD23	-1.0	protein involved in bud-site selection
NRG1	-1.6	binds to UAS-1 in the STA1 promoter and can interact with Ssn6; transcriptional repressor
UBC1	-1.2	ubiquitin-conjugating enzyme
SEM1	-2.6	Regulator of exocytosis and pseudohyphal differentiation
EMI1	-1.5	early meiotic induction
SPO73	-1.4	meiosis-specific protein of unknown function, required for spore wall formation during sporulation
SPR6	-1.3	sporulation-specific protein
ISC10	-1.7	meiosis-specific protein required for spore formation
ERV14	-0.9	14 kDa protein found on ER-derived vesicles
SPO12	-1.5	sporulation protein
SPO16	-1.0	sporulation protein
SSP1	-1.2	essential for meiosis and spore formation
YKL082C	-0.9	required for normal pre-rRNA Processing
DAD2	-1.3	outer kinetochore protein - part of Dam1 complex
BUD20	-1.8	protein involved in bud-site selection
ADY4	-0.9	component of the meiotic outer plaque
BUB2	-0.9	protein required for cell cycle arrest in response to loss of microtubule function
SAP30	-1.7	subunit of the histone deacetylase B complex
SPO1	-0.9	similar to phospholipase B
SPR1	-1.1	exo-1,3-beta-glucanase, sporulation-specific
YOR304C-A	-1.1	protein of unknown function; GFP-fusion protein localizes to the cell periphery, cytoplasm, bud, and bud neck
SPS4	-1.6	sporulation-specific protein
**Unclassfied proteins**		
YAL037W	-1.3	questionable protein
YAL044W-A	-1.0	similar to pombe uvi31, putative DNA repair protein
YAR023C	-1.3	putative integral membrane protein, member of DUP240 gene family
YAR069C	-1.0	questionable protein
YBL029C-A	-1.4	protein of unknown function; GFP-fusion protein localizes to the cell periphery
MOH1	-0.9	questionable protein
YBL059W	-1.1	questionable protein
YBL107C	-1.4	questionable protein
YBL108C-A	-1.1	questionable protein
YBR062C	-1.2	questionable protein
YBR262C	-2.1	protein localized to the mitochondria
YCL056C	-1.7	protein of unknown function; GFP-fusion protein localizes to the cytoplasm
YCR007C	-1.4	putative integral membrane protein, member of DUP240 gene family
YCR016W	-1.0	questionable protein
YCR022C	-1.1	questionable protein
YCR097W-A	-1.6	questionable protein
YCR102C	-1.2	questionable protein

Gene name	Fold change	Gene Product
YDL099W	-1.1	protein of unknown function; GFP-fusion protein localizes to the cytoplasm
YDL114W-A	-1.7	questionable protein
YDL121C	-1.2	questionable protein
YDL133W	-1.3	questionable protein
YDL156W	-1.1	questionable protein
YDL157C	-1.1	questionable protein
YDL159W-A	-1.1	questionable protein
YDL177C	-1.6	questionable protein
YDL240C-A	-1.4	questionable protein
YDL247W-A	-1.7	questionable protein
DOS2	-1.1	protein of unknown function, GFP-fusion protein localizes to the cytoplasm
SHU2	-1.7	suppressor of hydroxy-urea sensitivity
YDR210W	-1.4	protein of unknown function; GFP-fusion protein localizes to the cell periphery
BSC2	-1.3	transcript encoded by this ORF shows a high level of stop codon bypass
PHM6	-2.2	protein of unknown function, expression is regulated by phosphate levels
YDR336W	-1.0	questionable protein
YDR339C	-1.2	protein required for cell viability
YDR357C	-1.2	protein of unknown function; GFP-fusion protein localizes to the cytoplasm
BCP1	-0.9	questionable protein
YDR379C-A	-1.2	questionable protein
YDR381C-A	-2.1	questionable protein
NKP1	-1.3	non-essential kinetochore protein
CWC21	-1.2	component of a complex containing Cef1p, putatively involved in pre-mRNA splicing
YDR532C	-1.5	questionable protein
YEL018W	-1.2	stable stochiometric subunit of the NuA4 complex
YEL028W	-1.3	questionable protein
IES6	-1.7	protein that associates with the INO80 chromatin remodeling complex under low-salt conditions
YEL048C	-2.1	questionable protein
RMD6	-1.9	required for meiotic nuclear division
YEL073C	-0.9	questionable protein
YER030W	-1.5	questionable protein
YER039C-A	-1.1	questionable protein
YER071C	-0.9	protein of unknown function; GFP-fusion protein localizes to the cytoplasm
IES5	-1.1	protein that associates with the INO80 chromatin remodeling complex under low-salt conditions
YER093C-A	-1.3	questionable protein
YER121W	-1.7	questionable protein
YER137C	-1.7	questionable protein
YER182W	-1.2	protein localized to the mitochondria
YFR026C	-1.6	questionable protein
YFR043C	-1.1	questionable protein
YGL050W	-1.1	questionable protein
YGL080W	-0.9	protein localized to the mitochondria
YGL108C	-1.3	protein of unknown function; GFP-fusion protein localizes to the cell periphery
YGL220W	-1.4	questionable protein
YGR021W	-1.1	questionable protein
YGR035C	-1.8	protein of unknown function, potential Cdc28p substrate
YGR042W	-1.2	questionable protein
YGR153W	-1.0	questionable protein
YGR154C	-0.9	questionable protein
YGR168C	-1.3	questionable protein
YGR206W	-1.1	questionable protein
YGR243W	-1.9	protein localized to mitochondria

Gene name	Fold change	Gene Product
YHL013C	-1.0	questionable protein
YHL042W	-1.2	questionable protein
YHL045W	-1.2	questionable protein
FYV4	-0.9	protein of unknown function, required for survival upon exposure to K1 killer toxin
YHR132W-A	-0.9	questionable protein
YHR162W	-1.0	questionable protein
KRE27	-1.3	killer toxin resistant
YIL029C	-1.8	questionable protein
YIL089W	-1.0	questionable protein
YIL127C	-2.0	questionable protein
YIL158W	-1.5	questionable protein
YIL161W	-1.6	questionable protein
YIL165C	-1.4	questionable protein
SDL1	-1.7	L-serine dehydratase
YJL049W	-1.1	questionable protein
ICS3	-1.3	protein of unknown function
YJL118W	-0.9	questionable protein
YJL122W	-1.3	questionable protein
YJL156W-A	-1.3	questionable protein
YJR011C	-1.1	questionable protein
YJR012C	-1.1	protein required for cell viability
YAE1	-1.6	essential protein of unknown function
YJR079W	-1.0	questionable protein
YJR082C	-1.6	stable subunit of the NuA4 complex
YJR085C	-1.3	questionable protein
YKL018C-A	-1.4	questionable protein
YKL061W	-1.0	questionable protein
YKL133C	-1.0	questionable protein
YKL137W	-1.1	questionable protein
YKR075C	-1.5	questionable protein
YLL014W	-2.3	questionable protein
YLR036C	-0.9	questionable protein
YLR051C	-1.4	protein required for cell viability
YLR065C	-1.1	questionable protein
YLR073C	-1.4	questionable protein
YLR099W-A	-1.2	questionable protein
YLR104W	-1.5	questionable protein
YLR108C	-1.0	questionable protein
YLR112W	-1.4	questionable protein
YLR126C	-1.1	questionable protein
QRI5	-2.4	mitochondrial protein of unknown function
YLR211C	-1.2	questionable protein
YLR218C	-1.1	questionable protein
YLR254C	-1.2	questionable protein
TMA7	-1.9	protein putative involved in cytoplasmic ribosome function
YLR281C	-1.5	questionable protein
YLR346C	-1.6	protein of unknown function; expression regulated by PDR1
YLR363W-A	-1.5	questionable protein
YLR376C	-1.4	platinum sensitivity protein
YLR408C	-1.6	questionable protein
YLR456W	-1.5	questionable protein
YLR460C	-1.1	questionable protein
YML011C	-1.0	questionable protein
YML053C	-1.0	questionable protein
YML108W	-1.5	protein of unknown function localised to cytoplasm and nucleus
YML119W	-0.9	questionable protein

Gene name	Fold change	Gene Product
YMR003W	-1.3	questionable protein
YMR040W	-2.5	questionable protein
YMR071C	-1.1	integral membrane protein
YMR114C	-2.3	questionable protein
YMR132C	-1.3	questionable protein
ICY1	-1.0	Protein that interacts with the cytoskeleton and is involved in chromatin organization and nuclear transport
YNL024C	-1.4	putative S-adenosylmethionine-dependent methyltransferase of the seven beta-strand family
YNL046W	-1.9	questionable protein
YNL056W	-1.1	questionable protein
YNL100W	-0.9	questionable protein
YNL122C	-1.2	questionable protein
YNL149C	-1.2	protein required for cell viability
YNL157W	-1.1	questionable protein
YNL211C	-2.1	questionable protein
YNR004W	-0.9	questionable protein
YNR020C	-1.4	questionable protein
YNR024W	-2.1	questionable protein
YNR040W	-1.0	questionable protein
YOL014W	-1.5	questionable protein
YOR012W	-1.2	questionable protein
YOR013W	-1.4	questionable protein
YOR052C	-1.3	questionable protein
FYV12	-1.6	protein required for survival upon exposure to K1 killer toxin
IES4	-1.1	protein that associates with the INO80 chromatin remodeling complex under low-salt conditions
YOR252W	-1.5	questionable protein
YOR305W	-1.5	questionable protein
YOR318C	-1.2	questionable protein
YPL039W	-1.8	questionable protein
YPL071C	-1.9	questionable protein
YPL108W	-1.3	questionable protein
YPL144W	-0.9	questionable protein
YPL267W	-1.2	questionable protein
YPR074W-A	-2.2	questionable protein
