# Supplementary material for: Quercetin Protects Saccharomyces cerevisiae against Oxidative Stress by Inducing Trehalose Biosynthesis and the Cell Wall Integrity Pathway
Source: PLoS One. 2012 Sep 18;7(9):e45494. doi: 10.1371/journal.pone.0045494 (PMC3445532; doi:10.1371/journal.pone.0045494)
Supplement: Table S3 — Transcription factors with documented direct regulation of genes induced by exposure to quercetin. (PDF) [file pone.0045494.s003.pdf]

Table S3. Transcription factors with documented direct regulation of genes induced by exposure to quercetin.

Genes were analysed using YEASTRACT (www.yeastract.com; Monteiro *et al.*, 2008) software and grouped by transcription factors.

Transcription Factor	ORF/Genes	%*	%**	Function
Ste12p	CDC19 FLC2 SWH1 FUI1 IRA1 DUR1,2 SHM1 ILV6 GLK1 YCL042w YCL074w YCR061w ABP1 GPD1 STP4 SED1 SEC7 YDR170w-a ADR1 EFT2 PDR15 TOM1 YEL043w PRB1 BUD25 ZRG8 PTC2 CHD1 FRS2 HXK1 CWH41 PYC1 PIL1 SOL4 MGA1 ENO1 DED81 KSP1 SCH9 YHR214w-a BCY1 NEO1 SIM1 KGD1 YIL169c PAN1 PRY3 SCP160 URA2 SSC1 JSN1 CWP1 GFA1 APE2 EAP1 GAP1 ECM4 HSP104 GIS3 YPS1 PDC5 YLR162w GSY2 ECM38 FKS1 FMP27 DAK1 HXT2 MYO5 SSO2 RKR1 TPS3 APC1 YNL190w VNX1 IRA2 HPF1 STI1 DFG16 CYT1 MSA1 INP53 LSC1 YOR343w-a YOR343w-b GDH1 CAM1 SEC16 FAS2	39.0	4.2	Transcription factor that is activated by a MAP kinase signaling cascade, activates genes involved in mating or pseudohyphal/invasive growth pathways; cooperates with Tec1p transcription factor to regulate genes specific for invasive growth
Rap1p	CDC19 FLC2 FUI1 YBL053w SDS24 GLK1 YCL042w SRB8 GPD1 STP4 SED1 SSD1 EFT2 RVS167 PDR15 EMI2 YEL043w ZRG8 PTC2 RSP5 DNF1 FRS2 HXK1 SCY1 ADE5,7 YGR054w PIL1 SOL4 MGA1 ENO1 BCY1 NEO1 BBC1 MHP1 NET1 PRY3 URA2 JSN1 APE2 EAP1 HSP104 YBT1 GIS3 YLR162w ECM38 VRP1 STE23 CAR2 TSL1 YMR086w GAD1 YNL190w ZWF1 BNI1 PHO91 FPK1 CUE5 PYK2 FAS2	25.9	3.9	DNA-binding protein involved in either activation or repression of transcription, depending on binding site context; also binds telomere sequences and plays a role in telomeric position effect (silencing) and telomere structure
Sok2p	CDC19 FLC2 SWH1 FUI1 MEC1 SSE2 DUR1,2 YCL042w SRB8 GPD1 BSC1 YDL038c STP4 WHI4 NTH1 TPS2 SED1 KIN1 EFT2 NHX1 EMI2 PRB1 SEC3 ZRG8 HXK1 PYC1 PIL1 SOL4 MGA1 ENO1 SIM1 YIL169c MHP1 NET1 PRY3 CWP1 GAP1 ECM4 PDC5 FKS1 HXT2 ZWF1 PHO91 STI1 DFG16 WTM1 GDH1 CAM1 GIP3	21.5	4.7	Nuclear protein that plays a regulatory role in the cyclic AMP (cAMP)-dependent protein kinase (PKA) signal transduction pathway; negatively regulates pseudohyphal differentiation; homologous to several transcription factors
Fhl1p	CDC19 FUI1 TPS1 DUR1,2 SDS24 APE3 GLK1 YCL042w YCR061w TUP1 STP4 SED1 ADR1 EFT2 RVS167 PDR15 NPL3 YDR433w EMI2 YEL043w PRB1 ZRG8 PTC2 FRS2 PIL1 MGA1 ENO1 DED81 SIM1 MHP1 NET1 PRY3 MIR1 APE2 MMM1 YPS1 YLR162w VRP1 CAR2 TCB3 PYK2 GDH1 GIP3	18.9	4.9	Transcriptional activator with similarity to DNA-binding domain of Drosophila forkhead but unable to bind DNA in vitro; required for rRNA processing; isolated as a suppressor of splicing factor prp4

Sko1p	RPG1 AIM3 TPS1 GLK1 GPD1 BSC1 STP4 NTH1 TPS2 SED1 PDR15 ZRG8 KAP123 FRS2 HXK1 PYC1 PIL1 SOL4 MGA1 ENO1 RTC3 GRE3 SEC24 SIM1 SUC2 CWP1 EAP1 GAP1 ECM4 HSP104 DCS1 FKS1 STE23 DAK1 TSL1 MSC1 HXT2 RKR1 UBP15 ZWF1 STI1 GIP3	18.4	6.7	Basic leucine zipper (bZIP) transcription factor of the ATF/CREB family, forms a complex with Tup1p and Ssn6p to both activate and repress transcription; cytosolic and nuclear protein involved in osmotic and oxidative stress responses
Skn7p	CDC19 SSE2 BSC1 TPS2 SED1 PDR15 FRS2 HXK1 CHC1 PIL1 SOL4 MGA1 DED81 SSC1 MIR1 SDH1 GAP1 DPS1 YPS1 GSY2 FKS1 TPS3 LAT1 APC1 ZWF1 PHO91 ARG1 IRA2 HPF1 YOR343w-a YOR343w-b GIP3 GPH1	14.5	5.2	Nuclear response regulator and transcription factor, part of a branched two-component signaling system; required for optimal induction of heat-shock genes in response to oxidative stress; involved in osmoregulation
Yap6p	SWH1 SSE2 GPD1 BSC1 STP4 WHI4 SED1 EFT2 YEL043w BLM10 HXK1 ADE5,7 SOL4 MGA1 SEC24 SIM1 ATP2 GAP1 DPS1 TSL1 MSC1 ADE17 YMR196w RKR1 SRV2 WHI3 ZWF1 FPK1 ARG1 IRA2 HPF1 STI1	14.0	5.1	Putative basic leucine zipper (bZIP) transcription factor; overexpression increases sodium and lithium tolerance
Cin5p	CDC19 SSE2 DUR1,2 BSC1 STP4 WHI4 SED1 YEL043w SOL4 MGA1 SEC24 SIM1 NET1 PRY3 APE2 GAP1 MMM1 VRP1 MSC1 ADE17 GCV2 YMR196w ZWF1 ARG1 HPF1 STI1 DFG16 CYT1 WTM1	12.7	4.9	Basic leucine zipper transcriptional factor of the yAP-1 family that mediates pleiotropic drug resistance and salt tolerance; localizes constitutively to the nucleus
Tec1p	CDC19 FUI1 DUR1,2 YCR061w STP4 SED1 SEC7 EFT2 HXK1 PYC1 PIL1 MGA1 SIM1 URA2 CWP1 GFA1 APE2 GAP1 FKS1 TCB3 RKR1 ZWF1 SEC21 VNX1 ARG1 IRA2 DFG16 GDH1	12.3	4.9	Transcription factor required for full Ty1 epxression, Ty1-mediated gene activation, and haploid invasive and diploid pseudohyphal growth; TEA/ATTS DNA-binding domain family member
Yap5p	FLC2 DUR1,2 EMC1 YCL074w BPL1 WHI4 FRS2 ADE5,7 SOL4 MGA1 ENO1 RTC3 TAO3 MHP1 JSN1 GAP1 YLR162w DAK1 MSC1 ADE17 GCV2 YMR196w ARG1 HPF1 CYT1	11.0	5.2	basic leucine zipper (bZIP) transcription factor
Reb1p	WHI4 TOM1 ILV1 BLM10 RET2 YGR054w UBR1 ENO1 ECM29 THR1 SEC24 KGD1 NET1 GFA1 SDH1 VPS13 GIS3 YLR162w SEC61 GCV2 BNI1 PHO91 KRE5 FAS2	10.5	4.7	RNA polymerase I enhancer binding protein; DNA binding protein which binds to genes transcribed by both RNA polymerase I and RNA polymerase II; required for termination of RNA polymerase I transcription
Phd1p	CDC19 SWH1 FUI1 EMC1 GPD1 BSC1 STP4 EFT2 NHX1 PRB1 ZRG8 SOL4 MGA1 SIM1 YIL169c PRY3 HXT2 ZWF1 ARG1 IRA2 STI1 DFG16 WTM1 CAM1	10.5	4.6	Transcriptional activator that enhances pseudohyphal growth; regulates expression of FLO11, an adhesin required for pseudohyphal filament formation; similar to StuA, an A. nidulans developmental regulator; potential Cdc28p substrate

[*] % of genes regulated by the transcription factor, relative to the total number of up regulated genes
[**] % of genes regulated by the transcription factor, relative to the number of genes regulated by the same transcription factor

Reference

Monteiro, P.T., Mendes, N., Teixeira, M.C., d'Orey, S., Tenreiro, S., Mira, N., et al. (2008) YEASTRACT-DISCOVERER: new tools to improve the analysis of transcriptional regulatory associations in *Saccharomyces cerevisiae*. *Nucl Acids Res* 36: D132-D136.
