# Supplementary material for: Quercetin Protects Saccharomyces cerevisiae against Oxidative Stress by Inducing Trehalose Biosynthesis and the Cell Wall Integrity Pathway
Source: PLoS One. 2012 Sep 18;7(9):e45494. doi: 10.1371/journal.pone.0045494 (PMC3445532; doi:10.1371/journal.pone.0045494)
Supplement: Table S4 — Transcription factors with documented direct regulation of genes repressed by exposure to quercetin. (PDF) [file pone.0045494.s004.pdf]

Table S4. Transcription factors with documented direct regulation of genes repressed by exposure to quercetin.

Genes were analysed using YEASTRACT (www.yeastract.com; Monteiro *et al.*, 2008) software and grouped by transcription factors.

Transcription Factor	ORF/Genes	%[*]	%[**]	Function
Ste12p	ACS1 YAR023c HTA2 LDB7 LSM2 YBL029c-a RIB1 PRE7 MOH1 YBL059w YBL107c PAU9 HHF1 HHT1 GAL10 QDR3 NRG2 POL30 YMC2 YSY6 NTC20 YPT10 MRPL37 YCL056c CIT2 YCR007c SRD1 YCR038w-a YCR097w-a YCR102c APC11 YDL159w-a INH1 HEM3 YDL240c-a NRG1 RPC11 SNF11 SSS1 TAF10 CSN9 YDR210w HTA1 BSC2 PHM6 FCF1 SEM1 LSM6 YDR379c-a ADA2 MFA1 RPL27b UBC8 TIM9 IES6 TCA17 RMD6 SBH2 SPO73 RPS24a IES5 LSM4 YER137c LSM5 BUR6 ISC10 YER187w YPT1 LOC1 ULI1 RPL29 CGR1 AGA2 TYW3 ERV14 MTC3 YGR050c YGR131w YGR153w GTO1 QCR9 PEX21 FMP43 YGR251w PXR1 SNF6 YHL042w TIM10 RPL27a FYV4 VMA22 IPI1 SPL2 SPO12 LIN1 EST3 QDR1 FLX1 NIT1 YAP5 RPC17 PET130 PAM16 ASF1 RPS21b RPB4 RPA34 YJL156w-a CYC1 YJR079w RPL43b MRP17 KTI12 VPH2 MTR2 YKR012c JLP1 THI73 TEN1 TRX1 RFU1 BUD20 YLR099w-a YLR108c YLR112w ZRT2 SMD3 YLR164w PUS5 RPL37a YLR218c ADY4 NDL1 PDR8 SMD2 YLR281c RPS30a MRPL15 RPL38 YLR346c FBP1 LSM3 YLR456w ERV25 VPS71 YML053c KAR5 YMR114c SIP18 ICY1 TAF9 HOR7 URA10 HHF2 NCE103 YNL043c YNL046w MFA2 YNR071c YOL014w YOL106w INO4 ZEO1 BDS1 CIN5 TOM6 YOR052c ARF3 RKI1 ORT1 SPP2 MBF1 RRG7 SPS4 YOR318c ISU1 POC4 PPT2 ICY2 SNT309 RRP15	29.0	8.5	Transcription factor that is activated by a MAP kinase signaling cascade, activates genes involved in mating or pseudohyphal/invasive growth pathways; cooperates with Tec1p transcription factor to regulate genes specific for invasive growth

Rap1p	POP5 ACS1 HAP3 YBL028c YBL029c-a PRE7 MOH1 KTI11 NRG2 NTC20 HMLALPHA1 HMLALPHA2 MATALPHA2 MATALPHA1 IMG2 HMRA2 HMRA1 YCR097w-a APC11 YDL133w YDL187c NRG1 SSS1 YDR248c BSC2 PHM6 YDR336w ADA2 RPL27b TIM9 IES6 RMD6 YEL073c CHZ1 SPO73 RPS24a IES5 RPL23b LSM5 RPL22b YPI1 RPL29 QCR6 MTC3 YGR035c YGR050c YHL045w RPL27a SPO12 AIM20 DAL7 RPC17 YJL049w ICS3 MRPL49 ASF1 RPS21b RPA34 RPL39 NCE101 CYC1 APS2 RPL43b MCM22 TIM8 SOR1 SFT1 MRT4 RRP14 KTI12 RPS27a RPS21a LDB18 THI73 RFU1 BUD20 YLR104w YLR112w YLR164w RPL37a NDL1 RPS28b RPS30a MRPL15 RPL38 RPS29a TRM9 RPS18b YML053c MRPL44 YNL024c PGA2 GIM3 IGO1 ATX1 BIO3 ZEO1 HRT1 YSP3 YNG1 RKI1 RPS28a RPS30b FYV12 RPL33b MBF1 SPS4 EEB1 ISU1 POC4 DAP1 OYE3 ICY2 BRR1	18.2	7.6	DNA-binding protein involved in either activation or repression of transcription, depending on binding site context; also binds telomere sequences and plays a role in telomeric position effect (silencing) and telomere structure
Fhl1p	YBL028c PRE7 KTI11 NTC20 GRX1 CIT2 BUD23 YDL133w SNF11 BSC2 ADA2 RPL27b TIM9 IES6 SBH2 SPO73 RPS24a RPL23b YER137c LSM5 RPL22b RPL29 QCR6 VMA7 YGR021w YGR050c SNF6 RPL27a SRB2 YHR162w ICS3 ASF1 RPS21b RPL39 RPL43b SOR1 SFT1 RPS27a RPS21a TRX1 RFU1 BUD20 YLR112w RPL37a NDL1 RPS28b RPS30a RPL38 RPS29a LSM3 RPS18b YML053c MFT1 BUB2 ICY1 HOR7 HHF2 HHT2 ZEO1 RKI1 RPS30b FYV12 RPL33b SPS4 ISU1 POC4 ICY2	10.7	7.4	Transcriptional activator with similarity to DNA-binding domain of Drosophila forkhead but unable to bind DNA in vitro; required for rRNA processing; isolated as a suppressor of splicing factor prp4

* % of genes regulated by the transcription factor, relative to the total number of up regulated genes

** % of genes regulated by the transcription factor, relative to the number of genes regulated by the same transcription factor

Reference

Monteiro, P.T., Mendes, N., Teixeira, M.C., d'Orey, S., Tenreiro, S., Mira, N., et al. (2008) YEASTRACT-DISCOVERER: new tools to improve the analysis of transcriptional regulatory associations in *Saccharomyces cerevisiae*. *Nucl Acids Res* 36: D132-D136.
